# Implication of specific retinal cell-type involvement and gene expression changes in AMD progression using integrative analysis of single-cell and bulk RNA-seq profiling

**DOI:** 10.1038/s41598-021-95122-3

**Published:** 2021-08-02

**Authors:** Yafei Lyu, Randy Zauhar, Nicholas Dana, Christianne E. Strang, Jian Hu, Kui Wang, Shanrun Liu, Naifei Pan, Paul Gamlin, James A. Kimble, Jeffrey D. Messinger, Christine A. Curcio, Dwight Stambolian, Mingyao Li

**Affiliations:** 1 Department of Biostatistics, Epidemiology and Informatics, University of Pennsylvania Perelman School of Medicine, Philadelphia, PA 19104 USA; 2 Department of Chemistry and Biochemistry, The University of the Sciences in Philadelphia, Philadelphia, PA 19104 USA; 3 Departments of Ophthalmology and Human Genetics, University of Pennsylvania Perelman School of Medicine, Philadelphia, PA 19104 USA; 4 Department of Psychology, University of Alabama At Birmingham, Birmingham, AL 35294 USA; 5 Department of Information Theory and Data Science, School of Mathematical Sciences and LPMC, Nankai University, Tianjin, 30071 China; 6 Department of Biochemistry and Molecular Genetics, University of Alabama At Birmingham, Birmingham, AL 35294 USA; 7 Department of Computer and Information Science, University of Pennsylvania, Philadelphia, PA 19104 USA; 8 Department of Ophthalmology and Visual Sciences, University of Alabama At Birmingham, Birmingham, AL 35294 USA

**Keywords:** Retina, Data integration, Genome informatics

## Abstract

Age‐related macular degeneration (AMD) is a blinding eye disease with no unifying theme for its etiology. We used single-cell RNA sequencing to analyze the transcriptomes of ~ 93,000 cells from the macula and peripheral retina from two adult human donors and bulk RNA sequencing from fifteen adult human donors with and without AMD. Analysis of our single-cell data identified 267 cell-type-specific genes. Comparison of macula and peripheral retinal regions found no cell-type differences but did identify 50 differentially expressed genes (DEGs) with about 1/3 expressed in cones. Integration of our single-cell data with bulk RNA sequencing data from normal and AMD donors showed compositional changes more pronounced in macula in rods, microglia, endothelium, Müller glia, and astrocytes in the transition from normal to advanced AMD. KEGG pathway analysis of our normal vs. advanced AMD eyes identified enrichment in complement and coagulation pathways, antigen presentation, tissue remodeling, and signaling pathways including PI3K-Akt, NOD-like, Toll-like, and Rap1. These results showcase the use of single-cell RNA sequencing to infer cell-type compositional and cell-type-specific gene expression changes in intact bulk tissue and provide a foundation for investigating molecular mechanisms of retinal disease that lead to new therapeutic targets.

## Introduction

AMD is a leading cause of legal blindness worldwide. It affects over 10 million Americans^1^, twice the number affected by Alzheimer’s disease and equal to the total of all cancer patients combined^2^, and is expected to increase as the population ages. AMD primarily affects the macula^3^, a specialized region in the retina of humans and non-human primates. While there are short-term therapies available for one type of AMD, the underlying disease has no proven treatments, and vision loss is an eventual outcome for many individuals. While advances in retinal disease diagnostics have progressed rapidly, specific treatments for AMD directed at primary genetic or metabolic defects have progressed slowly due to a lack of understanding of the disease pathway. The slow progress is a result of multiple factors including lack of information about cell types involved in the initiation of AMD; anatomical and molecular differences between humans and commonly used laboratory animals; and inadequate supplies of postmortem human eyes to study pathophysiology.

**Figure 1:**
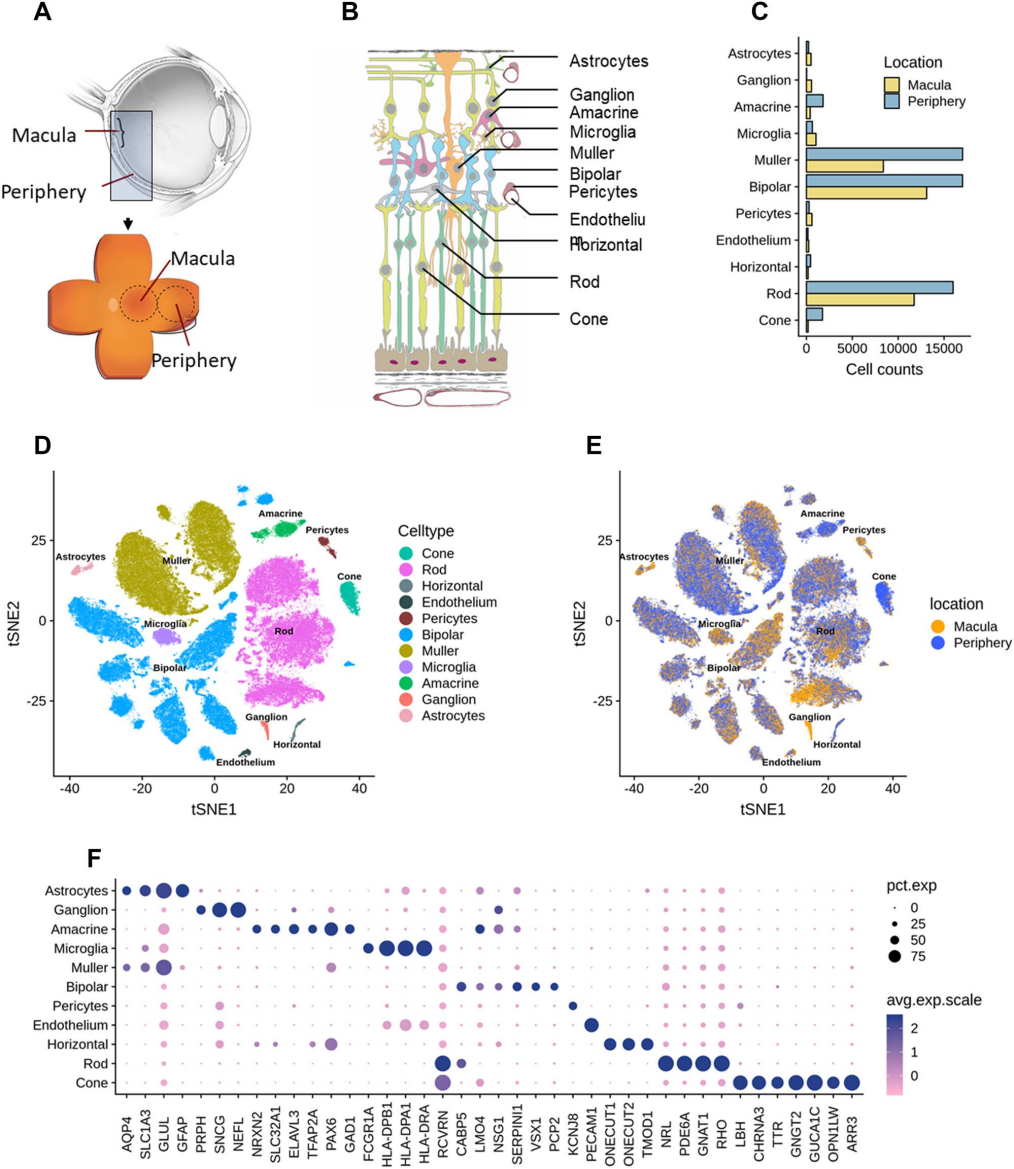
Summary of single-cell analysis from human retina. (**A**) Schematic cross-section of human eye (top) showing the retina lining the interior surface. The macula contains the fovea and is responsible for sharp vision. The periphery is responsible for detecting light and motion. Schematic of dissected tissue (bottom) shows retina adjoined to support tissues, flattened with relaxing cuts. Areas 8 mm in diameter were excised for RNA sequencing. (**B**) Layers of human retina and supporting tissues showing 11 assayed cell types. Five neuronal classes are photoreceptors, bipolar cells, ganglion cells, horizontal and amacrine cells. Cone photoreceptors are sensitive to color and bright light. Rod photoreceptors are sensitive to low light. Ganglion cells transmit information to the brain. Horizontal cells and amacrine cells modulate signal from photoreceptors and bipolar cells, respectively. Müller glia span the retina and are involved in neurotransmission, fluid balance, and wound repair. Also depicted are microglia (with phagocytic and immune activity), astrocytes (regulation of metabolism and blood brain barrier, synaptogenesis, neurotransmission), vascular endothelium (vascular tone and blood flow; coagulation and fibrinolysis; immune response, inflammation and angiogenesis) and pericytes (integrity of endothelial cells, trans-regulation of vascular tone, stem cells). The retinal layers include: NFL, nerve fiber layer; GCL, ganglion cell layer; IPL, inner plexiform layer; INL, inner nuclear layer; OPL, outer plexiform layer; ONL, outer nuclear layer; IS, OS, inner segments and outer segments of photoreceptors. Below the rods and cones are (from upper to lower) retinal pigment epithelium, Bruch’s membrane, and choriocapillaris, which are shown for completeness and were not assayed. (**C**) Bar plots showing proportions of counts of cells in each identified cell types from the scRNA-seq data across the two retina regions. Note that the counts of cells in each cell type do not reflect cell type composition in the tissue. (**D**) Visualization of scRNA-seq clusters from combined macula and periphery using t-SNE. Cells are colored by cell types. (**E**) Visualization of scRNA-seq clusters using t-SNE. Cells are colored by region of origin-macular or periphery. Note clusters are represented by both macula and peripheral regions. (**F**) Dot plots showing expression pattern of known gene markers across cell types (Supplementary data 1).

The human retina is composed of multiple layers, and each layer contains distinct cell types (Fig. 1A, B). There are 5 neuronal cell types in the retina that include photoreceptors, bipolar cells, ganglion cells, horizontal, and amacrine cells. Cone photoreceptors are sensitive to color and bright light. Rod photoreceptors are sensitive to dim light. Photoreceptors transmit information to bipolar cells, which in turn make synaptic contacts with ganglion cells. The axons of ganglion cells comprise the optic nerve and transmit information to the brain. Horizontal cells and amacrine cells modulate signals from photoreceptors and bipolar cells, respectively. A major glial cell, Müller, spans the retina and is involved in neurotransmission, fluid balance, and wound repair. Retinal neurons and their support cells form a highly organized, vertically integrated physiologic unit. AMD is a disease of this unit, with secondary effects including gliosis, cell death, and synaptic circuitry corruption^4–6^. There is an urgent need to identify the gene expression and cell type-specific changes that lead to AMD and accompany disease progression.

Regional differences exist in the retina and it is common to divide the retina into macula and peripheral regions due to differences in anatomical, cellular, molecular, and function^7–10^. Early publications of the retinal transcriptome were focused on describing the overall gene expression of the retina^11,12^ and later reporting the transcriptome differences between macula and periphery using bulk RNA sequencing (RNA-seq)^13^. While these studies described differentially expressed genes between macula and periphery, they lacked details of cell-type-specific expression. Cell-type-specific expression of the retina will help understand the biology of multiple diseases affecting the retina, beyond AMD.

Recent technologic breakthroughs in single-cell RNA sequencing (scRNA-seq) have made it possible to measure gene expression in single cells, resolve cell types, characterize the signature of gene expression across cells, and improve understanding of cellular function in health and disease. ScRNA-seq has been used to profile the transcriptome of retina cell types. Macosko et al. (2015) and Shekhar et al. (2016) generated scRNA-seq data from mouse retina and identified cell types and novel expression signatures^14,15^. Peng et al. (2019) profiled the cell types and gene expression changes for the macaque fovea (a cone-only sub-region of macula) and peripheral retina. There also have been publications on scRNA-seq of human retina which provided a resource for understanding human retina cell biology and diseases^16–21^. However, these studies focus on single-cell/nuclei data on non-AMD human retina tissues which cannot provide sufficient insights into the impact of AMD on retina expression pattern and cell-type composition. Further, several publications had long postmortem times (> 6 h)^16^ while others had low counts for nuclei or cells (< 25,000)^16,17,19,20^.

In the paper, we report the generation of a transcriptome atlas from scRNA-seq that contains 92,385 cells across two human retina regions, macula and periphery. Of particular note is our incorporation of short postmortem time. Long postmortem periods before processing of tissue (e.g. > 6 h) will affect RNA integrity and results of gene expression^22–24^. In addition, our study reaches beyond previous single-cell publications by correlating single-cell RNA expression with bulk tissue RNA expression of both control and AMD retina tissues. It is known that cell dissociation and capture steps are biased for certain cell types^25^, making it difficult to obtain accurate cell-type proportion estimates from scRNA-seq. To address this issue we used a novel deconvolution approach that integrates bulk RNA-seq and scRNA-seq to conduct cell-type-specific expression and cell-type composition analysis in intact bulk tissues, thus allowing the identification of cell-type-specific association of gene expression with AMD^26^. Our results revealed distinct cell-type-specific gene expression and cell-type composition changes associated with AMD progression and provided a framework for future studies incorporating bulk RNA-seq and scRNA-seq. In addition, our study identified additional novel cell-type-specific markers adding to the previous knowledge of specific retinal makers.

## Results

### Single-cell RNA-seq identified transcriptionally distinct clusters among neuronal and non-neuronal cells

Posterior eyecups from two Caucasian adult male donors, aged 78 and 90, were obtained from the Advancing Sight Network, Birmingham, Alabama (formerly the Alabama Eye Bank), within 6 h postmortem. Relief cuts were used to flatten the posterior eyecup and the macula was visualized under a dissecting microscope, which revealed no visible chorioretinal pathology. A sterile, RNAse-free 8 mm trephine was centered on the macula to collect the macular samples. After collection of the macular samples, a new sterile, RNAse-free 8 mm trephine was placed in the temporal region so that the inner edge of the second punch contacted the outer edge of macular punch. Retina was carefully separated from the underlying retinal pigment epithelium and choroidal vasculature (Fig. 1A, B) and dissociated according to published protocols^27^. Dissociated cells, 36,959 and 55,426 cells from macula and periphery were processed through a 10X Chromium platform (Supplementary Fig. 1a, b). Unsupervised deep learning-based clustering based on 2,000 highly variable genes identified 18 cell clusters, which were then assigned to neuronal and non-neuronal cell types by their canonical gene markers^28^. Two clusters could not be annotated due to a small number of cells (< 50) and were removed from further analysis resulting in 16 cell clusters. Then by collapsing cell clusters based on pairwise differential expression analysis (Supplementary Fig. 2), we obtained 11 major cell types (Fig. 1C, D, Supplementary Fig. 3). All identified cell types are found in both retinal regions and donors (Fig. 1E, Supplementary Fig. 1c). To validate the cell type annotation, we examined expression patterns of well-known retinal expression makers across the annotated cell types (Supplementary data 1). *GFAP* was selectively expressed in astrocytes; *PRPH*, *SNCG*, and *NEFL* marked ganglion cells; *NRXN2*, *SLC32A1*, *ELAVL3*, and *GAD1* were highly expressed in amacrine cells; *VSX1* and *PCP2* were selectively expressed in bipolar cells; *ONECUT1, 2* and *TMOD1* were selectively expressed in horizontal cells; *NRL*, *PDE6A*, *GNAT1*, and *RHO* were highly expressed in rods, and *CHRNA3*, *TTR*, *GNGT2*, *GUCA1C*, *OPN1LW*, and *ARR3* were selectively expressed in cones. *FCGR1A* was selectively expressed in microglia. *KCNJ8* was selectively expressed in pericytes; *PECAM1* was selectively expressed in endothelium.

**Figure 2:**
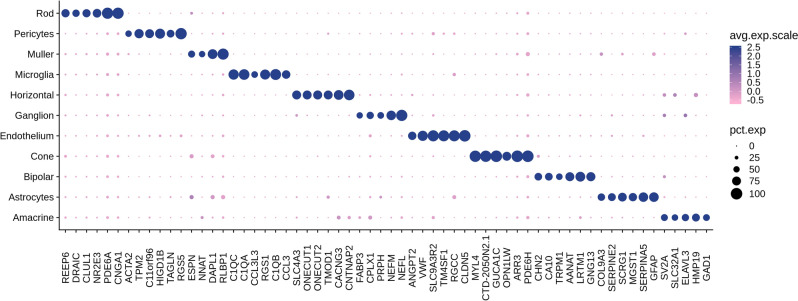
Dotplots showing the expression pattern of selected cell type specific markers across reina cell types. 6 top (ranked by percent of expression) specific markers for each retina cell type were selected and presented.

To identify cell-type gene markers that are specifically expressed in particular cell types, we performed differential expression analysis between each cell type vs. all other cells. In total, we identified 267 genes showing significant cell-type-specific expression (Methods, Supplementary data 2a). Figure 2 shows selected cell-type-specific markers for each cell type. Many of these findings were also reported in other scRNA-seq studies in retina. For example, some of the cell-type-specific expression detected in this study were previously reported to be enriched in rod (*PDE6A*, *CNGA1*), cone (*PDE6H, ARR3*), microglia (*CCL3, C1QA, C1QB*), endothelium (*RGCC, CLDN5*), amacrine (*GAD1*), ganglion (*NEFM*) and bipolar (*TRPM1, AANAT, LRTM1*)^17,19,29^. Though the cell-type-specific marker detection results may partially depend on the clustering algorithm resolution and sample preprocessing, we found that the top enriched markers for the major cell types are robust and reproducible across different studies. Considering the possible discrepancy of cell type markers between retina regions, we also identified cell-type-specific markers for macula and periphery separately, with 243 markers identified in macula while 282 markers identified in peripheral retina (Supplementary data 2b, c). By comparing the cell-type-specific markers identified in two retina regions for each cell type, we found that even though some markers identified in one retina region didn’t pass the cell type specificity criteria in the other region, they are still ranked high based on p-values from the differential expression analyses.

### Distinct expression pattern between human retina regions

Although the two human retina regions, macula, and periphery, share the same major cell types, their gene expression patterns exhibit regional specificity^3^. To characterize region specificity for each of the 11 major cell types, we performed differential expression analysis between the two retina regions, and 46 genes were found to be differentially expressed across all 11 cell types (Methods, Supplementary data 3). We found a relatively large number of differentially expressed genes (DEGs) in cones (n = 15). The numbers of DEGs detected for the other cell types are smaller: amacrine (n = 2), astrocytes (n = 6), bipolar (n = 1), endothelium (n = 2), horizontal (n = 1), microglia (n = 7), Müller cells (n = 9), pericytes (n = 2), and rods (n = 1). The variation in the number of DEGs between the two retina regions for different cell types is possibly due to the different levels of region-specificity in expression patterns across cell types. However, such variation may also be due to the different levels of cellular heterogeneity within each cell type.

Cone and rod cells are two types of photoreceptors in human retina that are responsible for vision at high and low light levels, respectively. The macula is a specialized area for vision with high spatial acuity. Further photoreceptors in the macula have long Henle fibers (axons). Thus, differences in expression patterns between macula and periphery are expected. For cone cells, we found *PCP4*, *RP11*, and *VTN* were highly expressed in the macular region, whereas *TTR*, *CLTB*, and *HSPB1* were preferentially expressed in the peripheral region. For rod cells, only *HSPA1A* was detected as a DEG between macula and periphery according to our criteria. Noticeably, *HSPA1A* also has consistently higher expression (fold change > 2) in macular region for four other neural cell types including amacrine, bipolar, and horizontal cells. For bipolar cells, we detected very few DEGs between macula and periphery, which is likely due to the transcriptional heterogeneity among the bipolar cells, as characterized by previous studies^3,14^.

### Single-cell RNA-seq identified transcriptionally distinct clusters among cone and bipolar cell neurons

Rod-mediated vision is affected early in AMD and cone-mediated vision can be preserved until late in the disease^30–32^. To further characterize these resilient cone cells and their connecting interneurons, we reclustered cones from the initial clustering. From this, we identified 4 distinct clusters for the cones based on long (L), medium (M), and short (S) wavelengths expression of *OPN1LW*, *OPN1MW*, and *OPN1SW*. One of the cone clusters has low expression across all known cone subtype markers, and it mainly expressed ribosomal protein, a sign of low-quality cells. Therefore, this cluster was removed from the following analysis as we were unable to assign it to any known cone subtypes. Due to small differences in 2 clusters, we combined long wavelength and medium wavelength cones and analyzed 2 major cone subgroups that contained S-cones and L/M-cones (Supplementary Fig. 4). Both cone subclusters showed differences in regional specificity: 34 DEGs were identified between macula and periphery for L/M-cones while 21 DEGs were detected for S-cones (Supplementary data 4a). Since recent studies have revealed multiple bipolar subtypes^3^, we also performed reclustering analysis on the bipolar cells. This analysis identified 14 distinct clusters that included rod bipolar cells (RBC), on-cone bipolar cells (OCBC), and off-cone bipolar cells (OFBC) based on their known bipolar markers (e.g., *PRKCA*, *LSL1*, and *GRM6*) (Supplementary Fig. 5a). Differential expression analysis of these 14 clusters identified 292 cluster-specific markers as well as DEGs between each pair of the subgroups (Supplementary data 4b, c). Expression patterns of the 14 bipolar subtypes in macula and periphery showed large differences between OFBC and RBC cells (Supplementary Fig. 5b-d). Reclustering of rods and Müller cells was not successful because we couldn’t find subpopulations that are biologically interpretable. As we increased the clustering resolution parameter, the rods and Müller cells started to separate by batch. As such, we didn’t pursue reclustering analysis further for these two cell types. Due to the small cell numbers, we didn’t perform reclustering analysis on astrocytes, microglia, and retinal ganglion cells.

### Cell-type level expression of AMD risk genes

**Figure 3:**
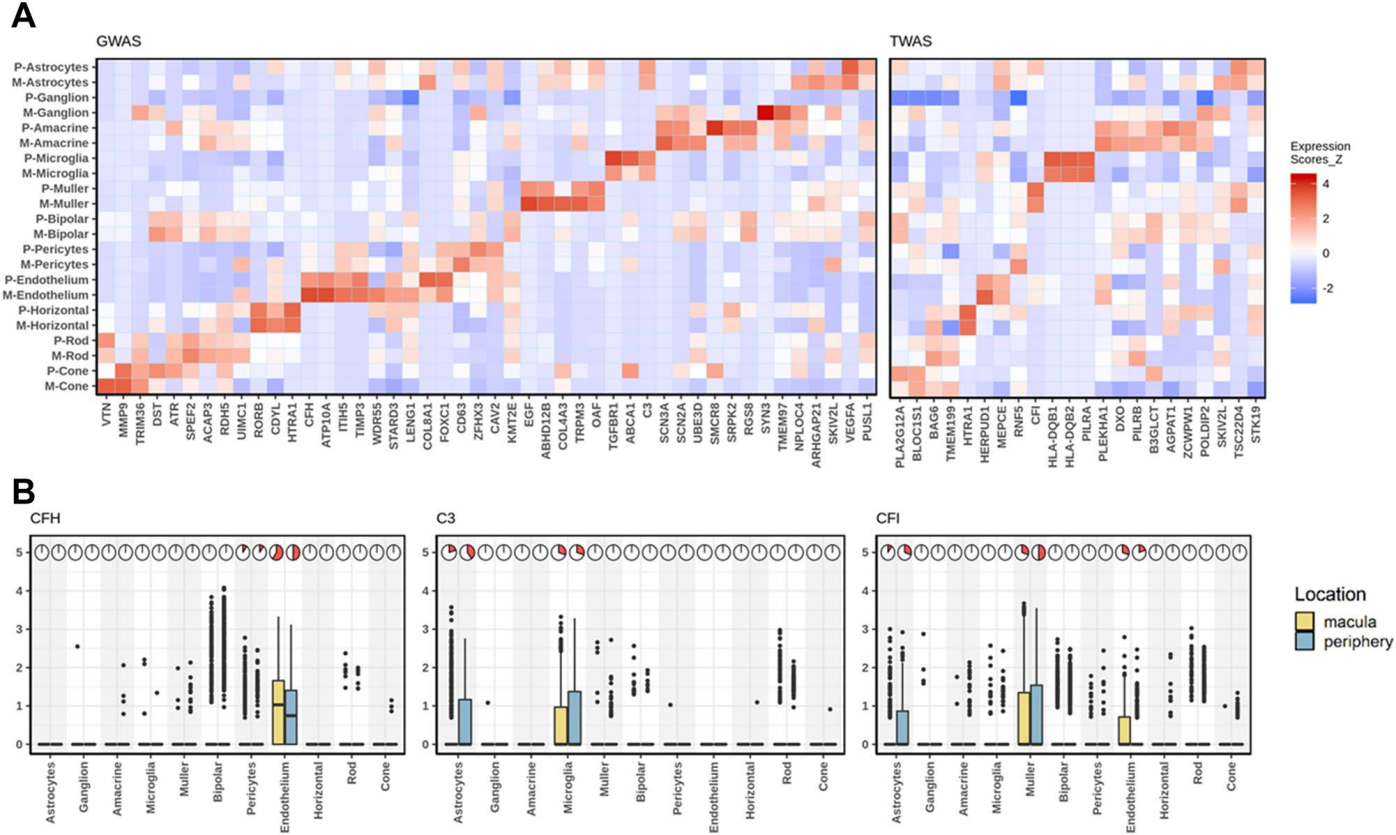
Cell type- and region-specificity of AMD risk genes. (**A**) Heatmap showing expression levels of AMD risk genes by cell type. Color in the heatmap represents expression intensity with red signifying higher expression in units of z-score. Left panel: AMD associated genes identified by loss- or gain-of-function mutations or by GWAS^3^. Right panel: target genes based on TWAS analysis listed^34^.Three AMD risk genes in the complement pathway, *CFH*, *C3* and *CFI*, were highlighted. (**B**) boxplot shows expression level of *CFH*, *C3* and *CFI* across cell types and retina regions. The pie chart show the percentage of cells expressing the gene in a particular region and cell type.

Next, we assessed the cell-type level expression of AMD risk genes identified from GWAS^33^ and transcriptome-wide association study (TWAS)^34^ (Fig. 3A, Supplementary Fig. 6). Of the 66 AMD risk genes, 23 were found to be among the 267 cell-type-specific genes. For example, *CFH* passed our stringent cell type specificity criterion (Methods) and it is specifically expressed in endothelium cells (Fig. 3B). The Y402H variant and other noncoding variants in *CFH* have been reported to be strongly associated with AMD, and the CFH protein acts as an inhibitor of the other complement cascade and has been localized in macular drusen by some groups but not others^35,36^. The relatively high expression level of *CFH* in endothelium suggests a role of endothelium cells in AMD. *CFH* is also specifically expressed in an on-cone bipolar subtype compared to other bipolar subtypes (Supplementary Fig. 5c). More than 80% of 431 cells (160 from macula and 271 from periphery) in this bipolar population expressed *CFH* gene. This bipolar subtype is featured by enriched expression of *CFH*, *SPOK3*, *NELL2*, and *TTYH1*. This *CFH* enriched-bipolar subtype needs to be further validated and investigated. Next, we examined cell type enrichment of two other AMD genes, *C3* and *CFI*. *C3* was preferentially expressed in microglia and astrocytes while *CFI* was expressed in astrocytes, endothelium, and muller cells (Fig. 3B). *C3* is a central complement component and a key inflammatory protein activated in AMD^37^. Its expression level is negatively regulated by *CFH* ^38^. However, such regulation is compromised in neurodegenerative diseases including AMD. Further, we found that the AMD risk gene *VTN* is differentially expressed between macular and peripheral cone cells (adjusted *P* < 0.05), indicating the potential regional difference of AMD impact.

### Differential expression analysis of bulk RNA-seq revealed differences in expression between early and advanced AMD

To investigate the impact of AMD on gene expression in human retina, we sequenced total RNA from macula and peripheral regions of 15 postmortem retinas that included control, early, and advanced AMD stages (Supplementary Fig. 7). The bulk RNA-data were generated from 13 macula samples (6 control, 4 early AMD, and 3 advanced AMD) and 15 periphery samples (8 control, 4 early AMD, and 3 advanced AMD) taken from the retina of the 15 adult donors. All donor eyes were collected within 6 h postmortem and characterized for presence of AMD and other pathology by author C.A.C. and a consulting medical retina specialist (J.A.K.) using ex vivo fundus imaging (color and optical coherence tomography, OCT). There is currently no consensus definition of AMD for clinical OCT imaging but AMD features are visible in post-mortem donor eyes^39^. By OCT, early AMD eyes are those with either drusen > 125 µm or roughening (or worse) of the RPE-basal lamina-Bruch’s membrane band, in the setting of drusen or subretinal drusenoid deposits. Control eyes lacked these features. Advanced AMD eyes had loss of outer retinal layers (OCT) and loss of pigmentation (color) in atrophy and in neovascular AMD, also exhibited subretinal fibrovascular scars. We identified 9,772 and 1,214 DEGs in macula and periphery, respectively, for advanced AMD vs. control comparison (Supplementary data 5a). A smaller number of DEGs between early AMD and control was found, with 169 DEGs in periphery and 21 DEGs in macula. We expected to see more DEGs in the macula than periphery and suspect that the larger sample size and higher sequencing depth of peripheral retina samples increased the power. Interestingly, we also found 17 DEGs for macula that may associate with AMD progression, as indicated by their increased fold change from early AMD to advanced AMD when compared to control.

To evaluate the implications of differential gene expression in the context of annotated genes and known biological pathways, we focused on genes with patterns of significant up- and down-regulation with respect to AMD disease stages. For both macula and peripheral retina, the significant DEGs for the early AMD vs. control comparisons were relatively few in number and did not identify significantly enriched KEGG pathways when submitted to the STRING database. For the advanced AMD vs. control comparison for macula, there were far too many genes to submit as a single query. Therefore, we constructed separate acceptable queries for up- and down-regulated genes by requiring a difference of fivefold or greater expression between control and advanced AMD, and also requiring that all genes in the queries have a minimum STRING connection weight of 0.5 or greater to at least one other gene in the query set (Methods). This protocol generated query lists of 1,905 up-regulated and 1,538 down-regulated genes for advanced AMD vs. control in macula (Supplementary data 5b). To offset the fewer number of DEGs in peripheral retina the fold-change criterion was reduced to twofold or greater with the previous requirement for minimum STRING connection weight still applied. We identified 449 up-regulated and 84 down-regulated genes for advanced AMD vs. control for peripheral retina (Supplementary data 5b). Coding genes from the lists were in turn used as queries against the KEGG database to select known biological pathways with significant overlap. KEGG pathways significantly enriched for our queries (adjusted *P* < 0.05) are listed in Supplementary data 6; genes up-regulated revealed pathways for complement and coagulation cascade, antigen presentation, and tissue remodeling when comparing advanced AMD vs. control for both macula and peripheral samples. Numerous signaling pathways are also up-regulated (PI3K-Akt, NOD-like, Toll-like, Rap1), in macula the TGF-beta pathway has been specifically suggested as a mechanism in AMD progression^40^. Genes down-regulated in the macula are enriched for pathways specific for neurons (Glutamatergic, GABAergic, Serotonergic, Cholinergic and Dopaminergic synapses, synaptic vesicle cycle, circadian entrainment) and phototransduction which is consistent with the significant loss of photoreceptors and other neuronal cell types in advanced AMD. For peripheral retina, our list of down-regulated genes did not identify any significantly-enriched pathways.

### The impact of AMD on retina cell-type composition

**Figure 4:**
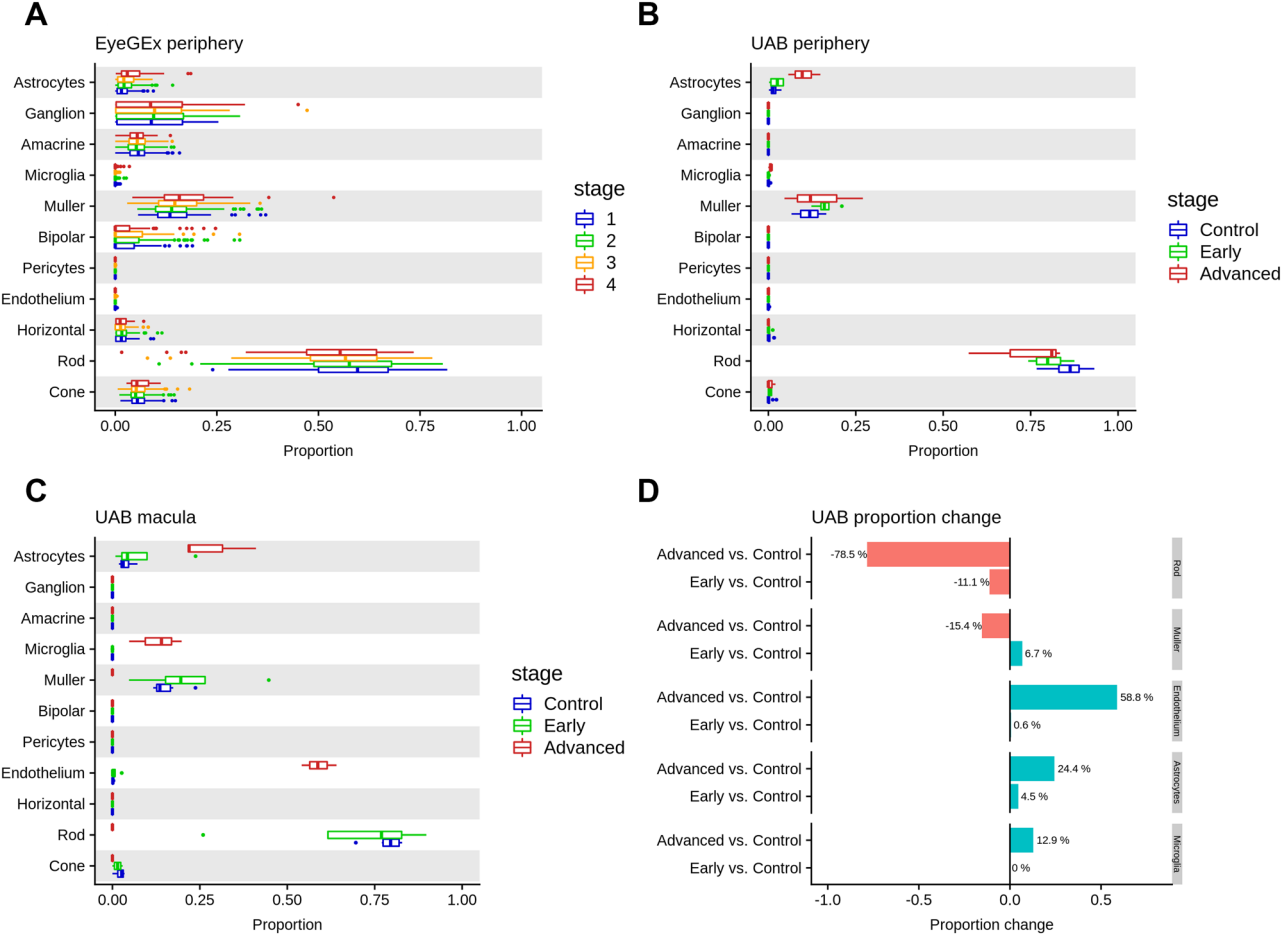
Cell-type deconvolution analysis from bulk RNA-seq data. Cell-type proportions for each bulk RNA-seq sample were estimated using MuSiC with the scRNA-seq data as reference. (**A**) Estimated cell-type proportions for the EyeGEx peripheral retina bulk RNA-samples with four stages of AMD (MGS1: 105; MGS2: 175; MGS3: 112; MGS4: 61). (**B**) Estimated cell-type proportions for the UAB peripheral retina bulk RNA-seq samples (control: 8; early AMD: 4; geographic atrophy, a advanced stage of AMD: 3). (**C**) Estimated cell-type proportions for the UAB macular retina bulk RNA-seq samples (control: 6; early AMD: 4; advanced AMD: 3). Note the similarity in (a) and (b) with respect to cell proportion increase in astrocytes and decrease in rods in peripheral retina as AMD progresses. Larger differences are noted in both cell types in macula along with additional increases in Müller glia, microglia and vascular endothelium as AMD progresses. (D) Cell-type proportion changes in the UAB macula retina samples for highlighted cell types.

Previous studies have shown that AMD has an impact on cell-type composition of the retina, particularly in macula^41,42^. Deconvolution is an analytical technique that can assess cell-type composition changes in bulk RNA-seq data using scRNA-seq as a reference^26^. We used deconvolution to analyze a large bulk RNA-seq dataset from the EyeGEx study^34^, which includes 453 RNA-seq samples from human peripheral retina. This study phenotyped eye samples using the Minnesota Grading System (MGS) (MGS1: 105; MGS2: 175; MGS3: 112; MGS4: 61) for AMD pathology in macula. Interestingly, we detected a significant cell-type proportion difference in astrocytes (adjusted *P* = 0.0043) between MGS1 (control) and MGS4 (more advanced stage) of AMD (Fig. 4A, Methods, Supplementary data 7). The increase in the proportion of astrocytes may reflect an immune response of the peripheral retina with AMD progression^43^. We also observed a tendency of increased median cell-type proportion for astrocytes and rods, as well as decreased median cell-type proportion for muller cells over disease progression.

Next, we tested our initial findings in another dataset, the UAB samples, which contain separate bulk RNA-seq for macula and peripheral retina from control and AMD donors. We performed cell-type deconvolution analysis in the peripheral retinas from our UAB sample and replicated the decrease in rods and increase in astrocytes with AMD progression, consistent with the EyeGEx data (Fig. 4B). The macular retina was also analyzed from these samples. The rods showed a slight decrease from control to early AMD and a more dramatic decrease from early to advanced AMD (Fig. 4C). Rods are barely detectable in the macula of advanced AMD (Control vs. Advanced adjusted *P* = 2.63E-6) (Fig. 4D and Supplementary Fig. 8, Supplementary data 7), which agrees with published histological evidence^41,44^. We also observed the tendency that endothelium, astrocytes, and microglia proportions increased in the macula with progression from control to advanced AMD.

### Cell-type-specific differential expression revealed genes associated with AMD

**Figure 5:**
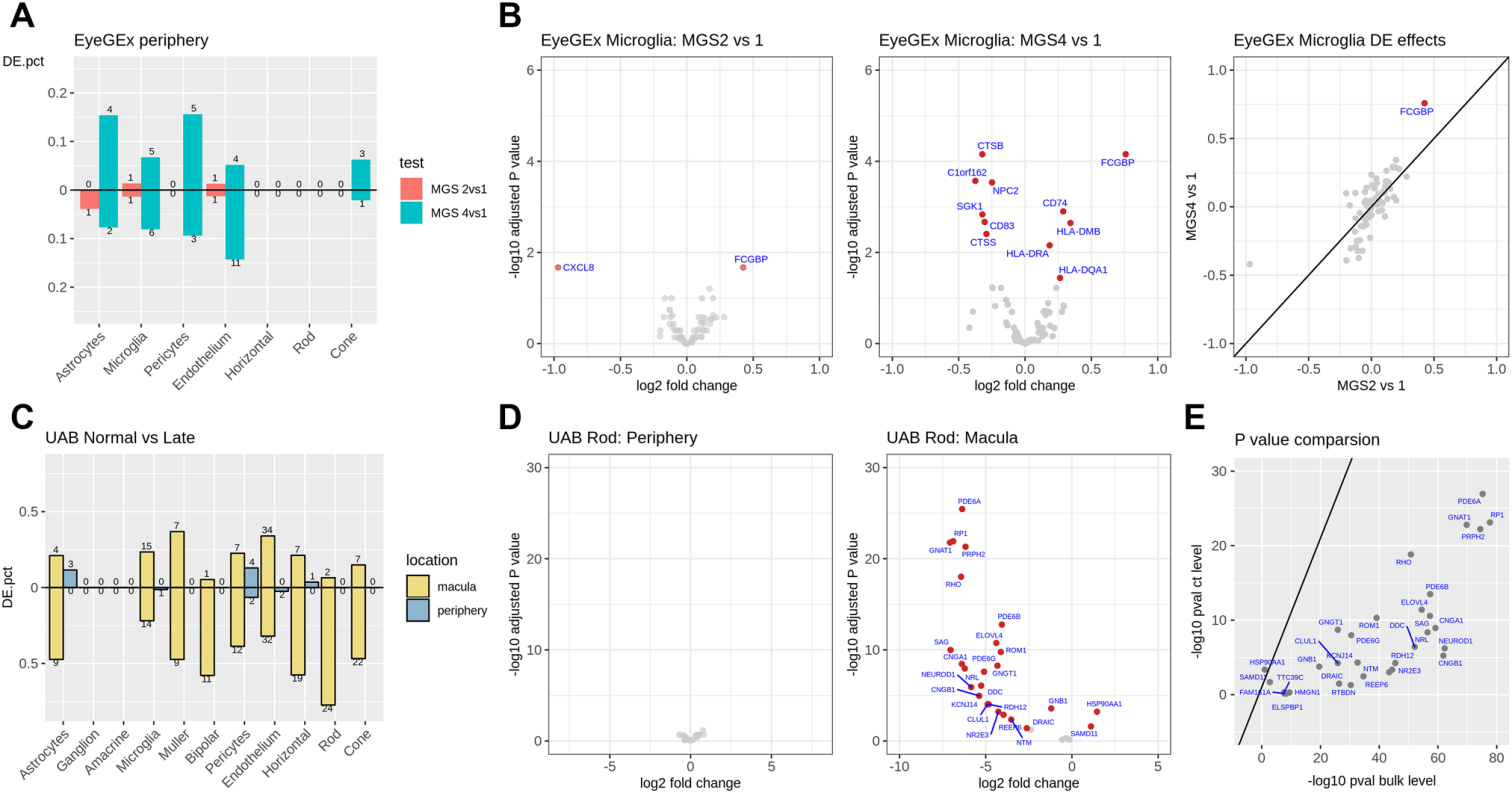
Cell type-specific differential expression analysis in two datasets. (**A**) Proportions (y-axis) of up- and down-regulated ctDEGs detected in the EyeGEx peripheral retina data. Colors show different test conditions: red for MGS2 vs. MGS1, and green for MGS4 vs. MGS1. Numbers above each bar indicate the number of detected ctDEGs for each comparison. (**B**) Volcano plots and effect size comparison of microglia-specific DEGs detected in the EyeGEx peripheral retina data. Significant ctDEGs were colored in red and annotated with gene names. (**C**) Proportions (y-axis) of up- and down-regulated ctDEGs for control vs. advanced AMD comparison in the UAB bulk RNA-seq data. Colors show different retina regions: blue for periphery, and yellow for macula. Numbers above each bar indicate the number of detected ctDEGs for each comparison. (**D**) Volcano plots and effect size comparison of rod-specific DEGs identified for control vs. advanced AMD comparison in the UAB bulk RNA-seq data. Significant ctDEGs were colored in red and annotated with gene names. (**E**) Comparison of p-values for cell type level and bulk level differential expression analysis for control vs. advanced AMD comparison in the UAB bulk RNA-seq data in macula rod cells.

Bulk RNA-seq measures the average expression of genes (sum of cell-type-specific gene expression weighted by cell-type proportions), therefore, bulk RNA-seq DEGs can be due to changes in cell-type-specific gene expression and/or cell-type composition. To determine if differential expression in the bulk RNA-seq samples was due to cell-type-specific differential expression and not cell-type composition, we developed a calibration-based method to detect cell-type-specific DEGs (ctDEGs) from bulk level gene expression for those cell-type-specific marker genes found in our scRNA-seq data (Methods). Applying this method to the EyeGEx peripheral retina data, we detected ctDEGs for each of the 11 major cell types. Comparing ctDEGs for different AMD stages MGS1, MGS2, and MGS4, we identified 5 ctDEGs for MGS2 vs. MGS1, and 44 ctDEGs for MGS4 vs. MGS1 (Supplementary Data 8). Microglia had a relatively large number of DEGs with 2 and 11 genes identified for the MGS2 vs. MGS1 and MGS4 vs. MGS1 comparisons, respectively (Fig. 5A). Notably, a microglia-specific DEGs, *FCGBP*, was detected in both MGS2 vs. MGS1 and MGS4 vs. MGS1 comparisons (Fig. 5B). This increased expression of *FCGBP* may suggest a microglia-specific AMD response with disease progression^45^.

Next, we applied the same analysis to the UAB bulk RNA-seq dataset and identified 5 ctDEGs in macula and 1 in periphery for control vs. early AMD (Supplementary Data 9). A larger number of ctDEGs were identified after comparing control and advanced AMD, with 13 ctDEGs found in periphery and 236 ctDEGs in macula (Fig. 5C). We compared the ctDEGs between the two retinal regions and found a considerable number of genes unique to each region (Fig. 5C). For example, we found expression of *RHO, RP1*, and *PDE6A* down-regulated in rod photoreceptors in AMD macula but not in periphery (Fig. 5D). *RHO* encodes a protein that is essential for vision in low-light conditions, *RP1* encodes protein that affects photosensitivity and outer segment morphogenesis of rod photoreceptors, while *PDE6A* involves visual signal transmission and amplification^46^. The down-regulated expression of these rod-specific genes reflects the compromised function of rod photoreceptors specific to macula of AMD eyes. These findings are consistent with the overall degeneration, dysfunction, and loss of rods. The few rods that were found are likely expressing fewer genes because they were degenerating, as expected. When comparing p-values of the ctDEGs with those obtained from bulk RNA-seq differential expression analysis (Fig. 5E), we found an attenuation of the p-value significance in ctDEGs because the bulk level analysis captures both gene expression and cell-type composition changes. This underscores the importance of delineating gene expression and cell type composition changes separately as is done in our analyses.

## Discussion

Advances in scRNA-seq technologies have revolutionized our understanding of cellular function in health and disease. Using scRNA-seq in human donor eyes, we constructed a high-resolution human retina cell atlas with a particular focus on the comparison of regional differences between macula and peripheral neurosensory retina. Our comprehensive analysis revealed 11 major cell types that are present in both retinal regions, a unique set of retinal cell type marker genes, and identification of the cell type modifications occurring in AMD. Further clustering analysis also identified subtypes for bipolar and cone cells and comparison between macula and peripheral gene expression revealed distinct gene expression patterns between regions. In total, we identified 267 genes that are cell-type-specific, expanding the previous datasets describing cell type-specific gene expression. Interestingly, vascular endothelium (93 genes) and microglia (42 genes) had over half of the 267 specific genes that were not expressed in any other cell types, suggesting that microglia have an important role in supporting the retina that cannot be compensated for by other cell types. The novel cell-type-specific marker genes identified in this study need to be validated using techniques such as immunohistochemistry or in situ hybridization. We will conduct such experiments in a separate study.

Our analysis of the impact of AMD on cell-type composition is particularly notable and speaks to the power of using single-cell data to decipher cell compositional changes in bulk RNA-seq data. Using the technique of deconvolution to integrate bulk RNA-seq data from donor retinas with and without AMD and scRNA-seq from normal retinas, we were able to infer cell-type proportions in the bulk RNA-seq samples which has not been attempted to date. It is notable that we observed cell-type compositional changes in select neurons and supporting cells, i.e. rods, Müller cells, vascular endothelium, astrocytes, and microglia (Fig. 4). Astrocytes, microglia, and vascular endothelium showed large proportional increases in the macula with advancing AMD and smaller increases in the periphery. The location of these dramatic increases is expected since AMD is known to adversely affect the macula more than the periphery. Rods, an early-affected cell type in AMD demonstrated a large proportional decrease in the macula and much smaller decrease in periphery from early AMD to the advanced GA stage^30^. Astrocytes, located mainly in the nerve-fiber and ganglion cell layers, defend the retina from damage through the reactive gliosis pathway which can be triggered by neuroinflammation and neurodegeneration. The hallmark of reactive astrogliosis is an increase and hypertrophy of astrocytes^47^. Retinas manifesting AMD have been reported to manifest reactive astrogliosis and our data support this result by demonstrating an increase in astrocyte activity in both early and advanced AMD retinas^43^ (Fig. 4B-D). Remarkably, this increase was present in both macula and peripheral locations, although greater in the macula region.

Müller glial cells span the width of the retina and perform a trophic function by supplying retinal neurons and photoreceptors with nutrients. Practically all retinal diseases are associated with an increase in activity of Muller cells^47,48^. Müller cell gliosis can be a double-edged sword, cytoprotective on retinal neurons in the early disease stage and cytotoxic in the later stage. Li et al. have exquisitely defined through histology the various activities of Müller cells in advanced GA stage including their enveloping cones into outer retinal tubulation and surviving the loss of photoreceptors in the outer nuclear layer^6^. Our results identified an increase of activity in Müller glia in the early AMD stage that could reflect their protective activities including clearing of drusen and release of neurotrophic factors and antioxidants^49^. In the advanced AMD stage, we found a decrease in Müller glia activity, possibly due to the massive gliosis^50^. Microglia are inactive under physiological conditions but become activated and acquire the ability to phagocytose and become neurotoxic leading to degeneration of photoreceptors during various retinal diseases, i.e. AMD^51^. In Fig. 4 it is apparent that microglia are quiescent during early AMD in the macula and periphery but manifest a significant increase in the macula of retinas affected with GA, as supported by recent histology^52^. This microglial increase in advanced AMD could be explained by a commensurate decline in the Müller glia population during this AMD stage. Müller glia expresses diazepam-binding inhibitor which regulates activation of microglia by limiting the magnitude of inflammatory response^53^. Therefore, the increased microglial response could be due to the loss of regulation secondary to the decline in Müller glia. Of course, this hypothesis would require further experiments for confirmation. Based on our results therapies for early AMD might be more successful if directed at protecting rods and Müller glia and inhibiting astrocyte activation. Such a conclusion was reached by Menon et al. using a different approach but similar conclusion^19^. They analyzed their scRNA-seq data for the ability of cell-type gene signatures to predict AMD genetic risk and found that Müller glia, astrocytes, microglia, and vascular endothelium were the most predictive of AMD risk. These authors also found that expression by cones was predictive.

Finally, we examined the cell type and region specificity of AMD risk genes reported in previous GWAS and TWAS studies^3,34^. Analysis of expression in our scRNA-seq data for 66 AMD risk genes identified 23 (35%) genes as cell-type-specific and 41 (62%) possessed differential expression between macula and periphery suggesting that gene expression differences between macula and peripheral retina may have functional relevance for the anatomical location in AMD. Unfortunately, we were not able to directly identify AMD associated genes at the cell type level due to the lack of scRNA-seq data from AMD eyes. Instead, we applied our deconvolution method to detect ctDEGs between control and AMD through the integration of bulk and scRNA-seq data. This procedure uncovered AMD associated DEGs at the cell type level across AMD stages and retina regions, which might have been masked at the bulk level. We observed that the AMD impact on cell-type-specific transcription landscapes varied between retina regions and increased along with disease progression.

In summary, we have constructed a high-resolution human retina cell atlas with a particular focus on the comparison of regional differences in the human retina. Our results linked GWAS genes for AMD with cell-type-specific gene expression and enabled the use of GWAS data to inform the genetic architecture of AMD. We further leveraged scRNA-seq and bulk RNA-seq data using an integrative analysis approach to reveal both cell-type composition as well as cell-type-specific gene expression changes associated with AMD progression. We also introduced novel methods for integrating scRNA-seq data with bulk RNA-seq data to elucidate the molecular mechanisms of disease in whole tissue. Our ongoing studies will aim to increase AMD sample size and add scRNA-seq data from the retinal pigment epithelium and choroid from both control and AMD eyes. This comprehensive approach will provide novel insights into cell-type-specific functions that will power precision therapeutics targeting AMD.

## Materials and methods

### Study subjects, scRNA-seq, and bulk RNA-seq for the UAB data

The scRNA-seq data were generated from macular and peripheral retina taken from two adult donors eyes lacking grossly visible chorioretinal pathology using the 10X Genomics Chromium™ system. The bulk RNA-data were generated from 13 macula samples (6 control, 4 early AMD, and 3 advanced AMD) and 15 periphery samples (8 control, 4 early AMD, and 3 advanced AMD) taken from the retina of 15 adult donors. All donor eyes were collected within 6 h postmortem and characterized for presence of AMD and other pathology in the ocular fundus by author C.A.C. and a consulting medical retina specialist (J.A.K.). All protocols were carried out following relevant guidelines and regulations as required by the UAB Institutional Review Board. Tissue collection protocols were approved by the institutional review board at the University of Alabama at Birmingham, complied with the Health Insurance Portability and Accountability Act of 1996, and adhered to the tenets of the Declaration of Helsinki. Informed consent was obtained by the Alabama Sight Network (ASN) from a legally authorized representative (LAR). Detailed sample preprocessing, donor characteristics, scRNA-seq and bulk RNA-seq data generation and preprocessing can be found in Supplementary Note.

### scRNA-seq data clustering and cell type identification

To identify cell types in the scRNA-seq data, we clustered cells into distinct cell types using DESC, a deep learning based clustering algorithm that is robust to batch effect^28^. To prepare the data for DESC clustering, the original gene count matrix obtained from CellRanger was normalized in which the UMI count for each gene in each cell was divided by the total number of UMIs in the cell. The normalized UMI count data were then multiplied by 10,000 and transformed to a natural log scale. We further standardize the log-transformed expression value for each gene by calculating a Z-score across cells within each batch. Lastly, 2,000 highly variable genes selected using *filter_genes_dispersion* function from the Scanpy package^54^ were used as input for DESC clustering. In DESC analysis, we used a 2-layer autoencoder with 64 nodes for the first layer and 32 nodes for the second layer. The DESC clustering was performed using a grid of resolutions, and resolution = 0.4 was selected because it yields high maximum cluster assignment probability for most of the cells. DESC initially identified 18 cell clusters and 16 of them that contain more than 50 cells were kept for downstream analyses. We annotated these 16 cell clusters with cell type labels by examining expression patterns of known retina cell type markers (Supplementary data 1). We further performed pairwise differential expression analysis among cell clusters, and confirmed that cell clusters with the same cell type annotation had few differentially expressed genes (Supplementary Fig. 2). This procedure resulted in 11 major neuronal cell types, including cone photoreceptors, rod photoreceptors, bipolar cells, horizontal cells, amacrine cells, and ganglion cells; support cells (microglia, Müller glia, and astrocytes), and vascular cells (endothelium and pericytes).

We are aware of that some of the cell types we identified, such as cone, rod, and ganglion cell, are commonly called cell classes^55^, since each of them includes multiple (sub) types of cells with different expression patterns. However, to simplify the analysis of neural and non-neural cells, we use cell type to signify both cell types and cell classes in our data.

### t-SNE visualization for scRNA-seq clustering

To visualize cell type clusters from the scRNA-seq data, we generated a two-dimensional non-linear embedding of the cells using t-distributed Stochastic Neighbor Embedding (t-SNE)^56^. The low denominational representation of the original data from DESC was used as input. The algorithm was implemented using the *mTSNE* function from python package MulticoreTSNE^57^. We set perplexity = 50 and learning rate = 500 and used the default values for all other parameters.

### Identification of cell-type-specific marker genes

To determine if a gene is preferentially expressed in a given cell type, we performed differential expression analysis to test whether a gene has a significantly higher expression in the given cell type than all other cell types. The analysis was implemented using the FindMarkers function in Seurat R package. We used the Wilcoxon test for the differential expression analysis by specifying test.use = "wilcox" and all other parameters were set as default. The *P* values were adjusted using Benjamini-Hochberg (BH) procedure and the significant (adjusted *P* value < 0.05, fold change > 2) DEGs from the test will be considered as candidates. Then, the genes which are widely expressed in the target cell type (percent of cell expressed the gene > 50%), but not in any of other cell types (percent of cell expressed the gene < 30%), were defined as cell-type-specific genes. We performed the cell type-specific markers identification by combining data from two retina regions, as well as using macula and periphery data separately. Identified cell-type-specific genes can be found in Supplementary data 2.

### Cell-type level differential expression between retina regions

To determine if a gene is preferentially expressed in a given retina region, for each cell type, we performed differential expression analysis to test whether a gene is differentially expressed between macula and peripheral region. The analysis was implemented using the FindMarkers function in Seurat R package. We used the Wilcoxon test for the differential expression analysis by specifying test.use = "wilcox" and all other parameters were set as default. The *P* values were adjusted using Benjamini-Hochberg (BH) procedure and the genes with adjusted *P* value < 0.05 and fold change > 2 were considered cell type level DEGs between retina regions. Identified cell type level DEGs can be found in Supplementary data 3.

### Cell-type level expression of AMD risk genes

We obtained AMD risk genes from previous studies, which include 51 AMD associated GWAS genes from Peng et al. 2019 ^3^ and 24 target genes identified from TWAS analysis by Ratnapriya et al.^34^. Genes that meet the following criteria were included for downstream analysis: 1) expressed in at least 1% of the cells; 2) expressed in at least 15 cells for at least one cell type in the scRNA-seq data. In total, 46 AMD associated genes and 20 TWAS target genes met these criteria. We searched these 66 AMD risk gene in identified cell-type-specific markers (Supplementary data 2) and found *CFH* is specifically expressed in endothelium cells.

To visualize the cell-type level expression of AMD risk genes, for each of them, we calculated the mean expression across cells for each of the 11 major cell types for macular and peripheral retina separately. To make cell-type-wise mean expressions comparable across genes, we calculated z-score of cell type mean expressions for each gene, and visualized the z-scores using heatmap (Fig. 3A).

### DEG detection in UAB bulk RNA-seq data

For the UAB data, we detected DEGs for macula and periphery separately. Genes that were expressed in less than 20% of the samples were eliminated, resulting in 19,313 genes in downstream analyses. The filtered read count matrices (19,313 genes by 13 samples for macula; 19,313 genes by 15 samples for periphery) were used as input. Then, the differential expression analysis was performed using DEseq2 (v1.22.2)^58^. For each retina region, we detected DEGs between control vs. early and control vs. advanced AMD. All parameters for DESeq2 were set as default. We used BH adjusted p-value < 0.05 as significance threshold. The significant DEGs are reported in Supplementary data 5a.

### Pathway analysis for DEGs detected in bulk RNA-seq data

The list of genes with significant differential expression between late AMD and control in macula retina was filtered to retain only genes with minimum fivefold change (either up- or down-regulated, late AMD / control). For peripheral retina, a lower threshold of twofold was applied. We further filtered the gene lists for macula and peripheral retina by removing ‘outlier’ genes with a STRING database interaction score of less than 0.5, using custom Python code, along with the file of score data downloaded from the database (https://stringdb-static.org/download/protein.links.detailed.v11.0.txt.gz). (‘Outlier’ genes, which include all non-coding genes, have limited or no representation in the aggregate STRING data, and do not meaningfully contribute to identifying annotated biological pathways.) The final sets of up- and down-regulated genes for macula and peripheral retina (Supplementary data 5b) were supplied as queries to the STRING database using its public web interface (https://string-db.org/), and the KEGG pathways with significant enrichment for the queries (adjusted *P* < 0.05) were downloaded from the website (Supplementary data 6).

### Cell-type deconvolution in bulk RNA-seq data

We performed cell type deconvolution analysis for both the EyeGEx and UAB bulk RNA-seq data using the UAB scRNA-seq data as the reference. For the scRNA-seq data, we only kept genes that were expressed in at least 5% of cells and more than 10 cells in at least one cell type. Cell type deconvolution analysis was conducted using MuSi ^26^ by setting eps = 0.0001, iter.max = 1,000 and default values for all other parameters. Also, we selected highly expressed genes in each cell type, totaling 1208 genes, as reference genes in the deconvolution.

To test the statistical significance of cell-type proportion changes estimated using EyeGEx data. We performed two-sample t-test between cell-type proportions estimated under MGS1 and MGS2, 3 and 4. The *P* values were then adjusted using BH procedure. The detailed result can be found in Supplementary Data 7. Similar tests were conducted in the UAB bulk RNA-seq data.

### DEG detection in EyeGEx bulk RNA-seq data

The Eye Genotype Expression (EyeGEx) study was designed to explore genetic landscape and post-GWAS interpretation of multifactorial ocular traits^34^. This study generated bulk RNA-seq data of 523 peripheral retinal samples from postmortem human donors. We obtained the EyeGEx bulk RNA-seq data from the Gene Expression Omnibus (accession number GSE115828). This dataset includes gene expression measures for 523 samples and 58,051 genes. 453 of the samples with AMD phenotype information (MGS1: 105; MGS2: 175; MGS3: 112; MGS4: 61) were included in the analysis^59^. Genes that were expressed in less than 20% of the samples were eliminated, resulting in 14,709 genes in downstream analyses. Then, the filtered RSEM count matrix (14,709 genes by 453 samples) was used as input. Differential expression analysis was performed between control vs. AMD samples defined by three different MGS levels using DEseq2 (v1.22.2)^58^. Also, to remove the potential batch effect and confounding factors, the following covariates were included in the analysis: sex, rna_isolation_batch, library_ preparation_batch, library_preparer, death_category, cholesterol, heart_disease, hypertension, rin and postmortem_interval_hrs. All parameters for DESeq2 were set as default. We used BH adjusted *P* value < 0.05 as the significance threshold. The DEG detection result from the EyeGEx data was then used in the cell type-specific DEG detection.

### Detection of cell type-specific DEGs in bulk RNA-seq using calibrated gene expression

Our analysis shows that AMD may have specific impact on particular cell types. We are interested in detecting genes that are differentially expressed between AMD and control eyes for different cell types separately. However, the bulk RNA-seq data with both AMD and control eyes lack cell type level information. To bypass such limitations, we developed a procedure to detect cell type-specific DEGs using bulk RNA-seq data calibrated by cell type proportion change between AMD and control eyes.

From bulk RNA-seq data, the fold change of gene expression between AMD and control eyes for gene *g* is

1 $$ FC_{g} = \frac{{\mathop \sum \nolimits_{{i \in S_{2} }} Y_{ig} /n_{2} }}{{\mathop \sum \nolimits_{{i \in S_{1} }} Y_{ig} /n_{1} }}, $$

where $Y_{ig}$ is the expression level of gene *g* in subject *i*, *S*_*k*_ is the set that includes all individuals in condition *k* (1 for control, and 2 for AMD), and *n*_*k*_ is the corresponding number of individuals in the set. Let $X_{ijg}$ be the expression level of gene *g* in subject *i* for cell type *j*, and *p*_*ij*_ be the proportion of cells from cell type *j* for subject *i*. The bulk RNA-seq expression can be written as weighted sum of cell-type-specific gene expression

2 $$ Y_{ig} = \mathop \sum \limits_{j = 1}^{C} p_{ij} X_{ijg} . $$

When gene *g* is cell type *j* specific, it is reasonable to assume that $Y_{ig} \approx p_{ij} X_{ijg}$ because the expression level of gene *g* in cell types other than *j* is low. This implies that

3 $$ FC_{g} \approx \frac{{\mathop \sum \nolimits_{{i \in S_{2} }} p_{ij} X_{ijg} /n_{2} }}{{\mathop \sum \nolimits_{{i \in S_{1} }} p_{ij} X_{ijg} /n_{1} }}. $$

To further simplify the computation, we assume cell type composition across subjects in the same condition are similar such that $p_{ij} = p_{j}^{k}$ for $i \in S_{k}$. Then by (3), we have

4 $$ FC_{g} \approx \frac{{p_{j}^{2} \mathop \sum \nolimits_{{i \in S_{2} }} X_{ijg} /n_{2} }}{{p_{j}^{1} \mathop \sum \nolimits_{{i \in S_{1} }} X_{ijg} /n_{1} }} = PC_{j} \cdot FC_{jg} , $$

where $PC_{j} = \frac{{p_{j}^{2} }}{{p_{j}^{1} }}$ is the proportion change of cell type *j* between AMD and control eyes, and $FC_{jg} = \frac{{p_{j}^{2} \mathop \sum \nolimits_{{i \in S_{2} }} X_{ijg} /n_{2} }}{{p_{j}^{1} \mathop \sum \nolimits_{{i \in S_{1} }} X_{ijg} /n_{1} }}$ is the fold change of gene *g* in cell type *j*. Taking log transformation on each side of (4), we have

5 $$ \log \left( {FC_{g} } \right) \approx \log \left( {PC_{j} } \right) + \log \left( {FC_{jg} } \right). $$

Let *G*_*j*_ be the set of genes that are cell type *j* specific, then

6 $$ \log \left( {PC_{j} } \right) \approx \frac{{\mathop \sum \nolimits_{{g \in G_{j} }} \log \left( {FC_{g} } \right)}}{{m_{j} }} - \frac{{\mathop \sum \nolimits_{{g \in G_{j} }} \log \left( {FC_{jg} } \right)}}{{m_{j} }}, $$

where *m*_*j*_ is the number of genes in *G*_*j*_.

If we assume that only few genes in *G*_*j*_ are differentially expressed, then their average fold change in log scale is approximately zero, which implies that

7 $$ PC_{j} \approx \exp \left( {\frac{{\mathop \sum \nolimits_{{g \in G_{j} }} \log \left( {FC_{g} } \right)}}{{m_{j} }}} \right). $$

Thus, *PC*_*j*_ can be estimated by the mean fold change of genes in *G*_*j*_ in log scale from the bulk RNA-seq data directly.

Although we can also estimate *PC*_*j*_ by cell type deconvolution results obtained from MuSiC, we have found the proportion change estimated this way is prone to outliers, which may result in large number of false positives in the detected ctDEGs. The estimated *PC*_*j*_ based on (7) is more robust in detecting ctDEGs by avoiding computational complexity introduced in deconvolution analysis.

Based on (3), we can calibrate gene expression contributed by cell type proportion change in AMD subjects by $Y_{ig}^{^{\prime}} = PC_{j} \times Y_{ig}$. This calibrated expression can be directly compared with gene expression in the control subjects to determine if gene *g* is differentially expressed in cell type *j*. With the calibrated gene expression in AMD subjects, we can perform differential expression analysis using DEseq2^58^ for genes preferentially expressed in a given cell types. To determine if a gene is preferentially expressed in a given cell type, we performed differential expression analysis to test whether a gene has a significantly higher expression in the given cell type than all other cell types. The analysis was implemented using the *FindMarkers* function in Seurat R package. We used the Wilcoxon test for the differential expression analysis by specifying test.use = "wilcox" and all other parameters were set as default. The significant (adjusted *P* value < 0.05, fold change > 2) genes from the Wilcoxon test were considered as candidates. Then, the candidate genes which are widely expressed in the target cell type (percent of cells expressing the gene > 50%) were tested for ctDEGs. Note that it is possible that a gene is specific to multiple cell types, although such cases are rare. We performed the ctDGE identification by combining data from two retina regions, as well as using macula and periphery data separately. All parameters in DESeq2 were set at default and genes with Benjamini-Hochberg (BH)^60^ adjusted p-value < 0.05 were declared to be significant. The detected cell type-specific DEGs are reported in Supplementary data 8 and 9.

## Supplementary Information

Supplementary Information 1.

Supplementary Information 2.

Supplementary Information 3.

Supplementary Information 4.

Supplementary Information 5.

Supplementary Information 6.

Supplementary Information 7.

Supplementary Information 8.

Supplementary Information 9.

Supplementary Information 10.

Supplementary Information 11.

Supplementary Information 12.

Supplementary Information 13.

Supplementary Information 14.

Supplementary Information 15.

Supplementary Information 16.

Supplementary Information 17.

Supplementary Information 18.

Supplementary Information 19.

Supplementary Information 20.

## Data Availability

The RNA-seq data reported in this paper can be downloaded from GEO (GSE155154 for UAB bulk RNA-seq, and GSE155288 for single-cell RNA-seq).
