# Supplementary material for: Implication of specific retinal cell-type involvement and gene expression changes in AMD progression using integrative analysis of single-cell and bulk RNA-seq profiling
Source: Sci Rep. 2021 Aug 2;11:15612. doi: 10.1038/s41598-021-95122-3 (PMC8329233; doi:10.1038/s41598-021-95122-3)
Supplement: Supplementary file 19 — Supplementary Information 19. [file 41598_2021_95122_MOESM19_ESM.docx]

**Supplementary**

**Implication of specific retinal cell-type involvement and gene expression changes in AMD progression using integrative analysis of single-cell and bulk RNA-seq profiling**

Yafei Lyu^1†^, Randy Zauhar^2†^, Nico Dana^3^, Christianne E. Strang^4^, Jian Hu^1^, Kui Wang^1,5^, Shanrun Liu^6^, Naifei Pan^7^, Paul Gamlin^8^, James A. Kimble^8^, Jeffrey D. Messinger^8^, Christine A. Curcio^8^, Dwight Stambolian^3*^, Mingyao Li^1*^

^1^Department of Biostatistics, Epidemiology and Informatics, University of Pennsylvania Perelman School of Medicine, Philadelphia, PA 19104, USA; ^2^Department of Chemistry and Biochemistry, The University of the Sciences in Philadelphia, Philadelphia, PA 19104, USA; ^3^Dept of Ophthalmology and Human Genetics, University of Pennsylvania Perelman School of Medicine, Philadelphia, PA 19104, USA; ^4^Department of Psychology, University of Alabama at Birmingham, Birmingham, AL 35294, USA; ^5^Department of Information Theory and Data Science, School of Mathematical Sciences and LPMC, Nankai University, Tianjin 30071, China; ^6^Department of Biochemistry and Molecular Genetics, University of Alabama at Birmingham, Birmingham, AL 35294, USA; ^7^Department of Computer and Information Science, University of Pennsylvania, PA 19104, USA; ^8^Department of Ophthalmology and Visual Sciences, University of Alabama at Birmingham, Birmingham, AL 35294, USA.

^†^ Equal contribution

**^*^** Correspondence to [mingyao@pennmedicine.upenn.edu](mailto:mingyao@pennmedicine.upenn.edu) or [stamboli@pennmedicine.upenn.edu](mailto:stamboli@pennmedicine.upenn.edu)

**Supplementary Note 1. scRNA-seq sample processing and data generation**

**Eye collection protocol.** Donor eyes from two Caucasian adult male donors, aged 78 and 90, were obtained from the UAB within 6 hours postmortem. Neither donor had a known history of retinal disease, head or ocular trauma, significant refractive error, neurological disease, diabetes, or uncontrolled hypertension.

**Dissection and dissociation of retina.** Following removal of the anterior chamber and vitreous, the eyecup was immersed in oxygenated Ames media. Relief cuts were made in the posterior eyecup to expose the retinal tissue and 8-mm-diameter punches were obtained from the macula and temporal periphery. Neurosensory retina was carefully isolated from the underlying retinal pigment epithelium and choroidal vasculature. The isolated retina tissue was dissociated with activated papain (Worthington Biochemical Corp.) as previously optimized to obtain a high percentage of viable retinal cells(*41*). After dissociation, magnetic bead-based removal of dead cells (Miltenyi Biotech) was used to reach the optimum target for viability of 85-95% per sample. Viability was determined by FACS sort or by staining an aliquot of the dissociated cells with trypan blue, 0.4% (Sigma-Aldrich).

**Single cell transcriptome library preparation and sequencing.** Single cell transcriptome libraries were prepared by using 10xGenomics Single Cell 3’ biased v2 kit according to the company’s manual. The constructed single cell libraries were sequenced by HiSeq 2000 sequencer (Illumina, Inc., San Diego, CA, USA) with total reads per cell targeted for a minimum of 50,000.

**Preprocessing of scRNA-seq data.** For each sample replicate, we performed initial quality control using Cell Ranger (Version 2.1.0). For retina macula samples, we initially obtained 33,694 genes by 48,800 cells count matrices. For macula periphery samples, we obtained 33,694 by 59,222 cells count matrices. Then, we further filtered the data using Seurat (version 2.3.4)(*38*). A cell was retained in downstream analyses if it meets the following criteria: (1) More than 200 genes are detected; (2) The proportion of the transcript counts from mitochondrial encoded genes is less than 25%; (3) Total number of UMIs is between 500 and 10,000. This resulted in 33,694 genes across 92,386 cells (macula: 36,959; periphery: 55,426), which were used for analysis shown in **Supplementary Fig. 1**.

**Supplementary Note 2. scRNA-seq data clustering and cell type assignment**

To identify cell types in the scRNA-seq data, we clustered cells into transcriptionally similar groups using DESC(*33*) (**Methods**). Initially, we obtained 18 cell clusters but decided to continue with16 cell clusters because 2 cell clusters had less than 50 cells. We annotated these 16 cell clusters with cell type labels by examining expression patterns of known retina cell type markers (**Supplementary Data 1**). We identified six bipolar subtypes. To examine these 16 cell clusters further, we performed pairwise differential expression analysis among 16 cell clusters using *FindMarkers* from the Seurat package. We enabled the Wilcoxon test by specifying test.use = "wilcox" and all other parameters were set as default, and used adjusted p-value<0.05, fold-change>2 as threshold to determine significant DEGs between each pair of cell clusters. We detected a considerable number of DEGs between each pair of cell clusters, except the pairwise analysis among the 6 cell clusters labeled as bipolar (**Supplementary Fig. 2**). Therefore, we maintained these 6 clusters as bipolar cell subtypes. In total, we determined 11 major cell types. **Fig. 1f** shows the expression patterns of representative known cell type markers across the 11 identified cell types.

**Supplementary Note 3. Identifying sub cell types for cone and bipolar**

**Identifying cone subtypes.** We identified cell subtypes from 1,942 cone cells be performing DESC(*33*) (**Methods**). The clustering result revealed 4 groups of cone cells (**Supplementary Fig. 4a**). We then performed differential expression analysis between cells in each group vs. all other cells as well as each pair of cell groups. We noticed that one of the cone cell clusters has low expression across all known cone subtype markers, and it mainly expressed ribosomal protein, a sign of low-quality cells. This group was considered as contamination (Cone_other) and it was removed from the following analysis. For the remaining 3 groups, we combined two of them since there are very few DEGs (n=2) between the two groups. At last, we labeled the cell groups as S-cone (Cone_s) and L/M-cone (Cone_LM) by looking at the known cone subtype markers, including OPN1SW and OPN1LW (**Supplementary Fig. 4b**).

**Identifying bipolar subtypes.** We identified cell subtypes from 30,126 bipolar cells by performing DESC(*33*) (**Methods**). The clustering result revealed 16 groups of bipolar cells (**Supplementary Fig. 5a**). We then performed differential expression analysis between cells in each group vs. all other cells as well as each pair of cell groups. We then combined 3 cell groups with less than 10 DEGs between each pair of them. We also assigned these cell groups into 3 main bipolar subtype categories: off cone, on cone and rod bipolar cell (RBC) by looking at the expression levels of known markers for each of the categories (**Supplementary Fig. 5b**). We further identified bipolar subtype markers (**Supplementary Fig. 5c**) and between retina region differential expression for each of the identified subtypes (**Supplementary Fig. 5d**).

We also attempted to investigate rod and muller subtypes and their region specificity but were not successful in separating cells into subpopulations.

**Supplementary Note 4. UAB bulk tissue processing and data generation**

**Histopathological analysis of UAB samples.** The Institutional Review Board at UAB approved the use of human tissues in this study. This study utilized 15 pairs of eyes from non-diabetic Caucasian donors 69-95 yr of age (84.73 yr ± 5.53 yr; 8 males and 7 females) at a death-to-preservation interval of < 6 hr. Ocular health histories were not available. Eyes were opened by eye bank recovery personnel using an 18 mm diameter corneal trephine, followed by a snip to the iris to facilitate penetrate of preservatives into the fundus. Preservatives were RNAlater (Qiagen) for the Left Eye and 2% glutaraldehyde and 1% paraformaldehyde in 0.1M phosphate buffer for the Right Eye, both at 4°C. Left Eyes were shipped on wet ice via overnight courier to University of Pennsylvania where they were processed upon arrival.

Maculopathy status of Right Eyes was assessed at UAB by a 3-component protocol. Eyes underwent multimodal ex vivo imaging of excised 8-mm diameter macular punches using digital color photography and spectral domain optical coherence tomography volume scans (SD-OCT; Spectralis, Heidelberg Engineering) with a custom tissue holder (co-author JDM). They also underwent internal globe examination using a dissecting scope (Nikon SMZ-U) with oblique trans- and epi-illumination in consultation with an MD medical retina specialist (co-author JAK). Finally, eyes were submitted for histopathology using macula-wide high-resolution sections. Macular punches including retina, RPE, choroid, and sclera were then post-fixed in osmium tannic acid paraphenylenediamine to accentuate neutral lipid-rich lesions associated with AMD (*42*, *43*). Sections 0.8 µm in thickness through the rod-free foveola and the rod-rich perifovea at 2000 µm superior to the fovea were stained with toluidine blue, examined, and annotated.

The definition of AMD used in this study(*44*) was the presence of one large druse (>125 µm in diameter) in the macula or severe RPE changes in the setting of at least one druse or continuous basal linear deposit, with or without the presence of neovascularization and its sequelae. Eyes with geographic atrophy had at least one region 250 µm in diameter lacking a continuous RPE layer (but possibly containing ‘dissociated’ RPE(*45*). Unremarkable eyes were those lacking characteristics of AMD or other chorioretinal disease as discernable in either histology or ex vivo imaging; these served as comparison eyes.

The use of fellow eyes optimally preserved for RNA-seq and for histopathology in this study is a limitation, because two eyes of one individual may be at different disease severity. We feel that this limitation is manageable, because it was recently found in a population-based cohort that was observed for 20 years, AMD severity in one eye was found to largely track AMD severity in the fellow eye at all stages of the disease(*46*). This published study also found a <10% chance of lifetime occurrence of asymmetry >2 steps on the grading scale for color fundus photography.

**Dissection and dissociation of retina.** Macula and periphery were dissected from retina resulting in two samples per eye. The tissues were isolated at the macula and periphery using a circular 10-mm biopsy punch.

**UAB data library preparation and sequencing.** RNA for the eye tissues was extracted using the AllPrep DNA/RNA Mini Kit (Qiagen). Extracted RNA samples underwent quality control assessment using R6K Screen Tape on a 2200 Tape Station (Agilent, Santa Clara, CA, USA) and were quantified using Qubit 2.0 Fluorimeter from Life Technologies (Grand Island, NY). All RNA samples selected for sequencing had an RNA integrity number of ≥8. Strand-specific RNA library was prepared from 100 ng totalRNAusing the Encore Complete RNA-seq library kit (Nugen Technologies, Inc., San Carlos, CA, USA) according to the manufacturer’s protocol. RNA sequencing was performed at the Center for Applied Genomics at the Children’s Hospital of Philadelphia per standard protocols. The prepared libraries were clustered and then sequenced using HiSeq 2000 sequencer (Illumina, Inc., San Diego, CA, USA) with four RNA-seq libraries per lane (2×101-bp paired-end reads).

**Preprocessing of bulk tissue data.**

The RNA-seq data were aligned to the hg38 reference genome using GSNAP (version 2016-06-30) with known splice sites (SNP file build 147) taken into account. In order to eliminate mapping errors and reduce potential mapping ambiguity owing to homologous sequences, several filtering steps were applied. Specifically, we required the mapping quality score of ≥30 for each read, reads from the same pair were mapped to the same chromosome with expected orientations and the mapping distance between members of the read pair was 200,000 bp. Quality control analysis of the aligned data was performed using program RNA-seQC. All subsequent analyses were based on filtered alignment files. Per-gene counts were generated from the GSNAP alignments using the HTSeq-count program (version 0.6.0) using default ‘union’ mode and the HG38 reference genome.

**
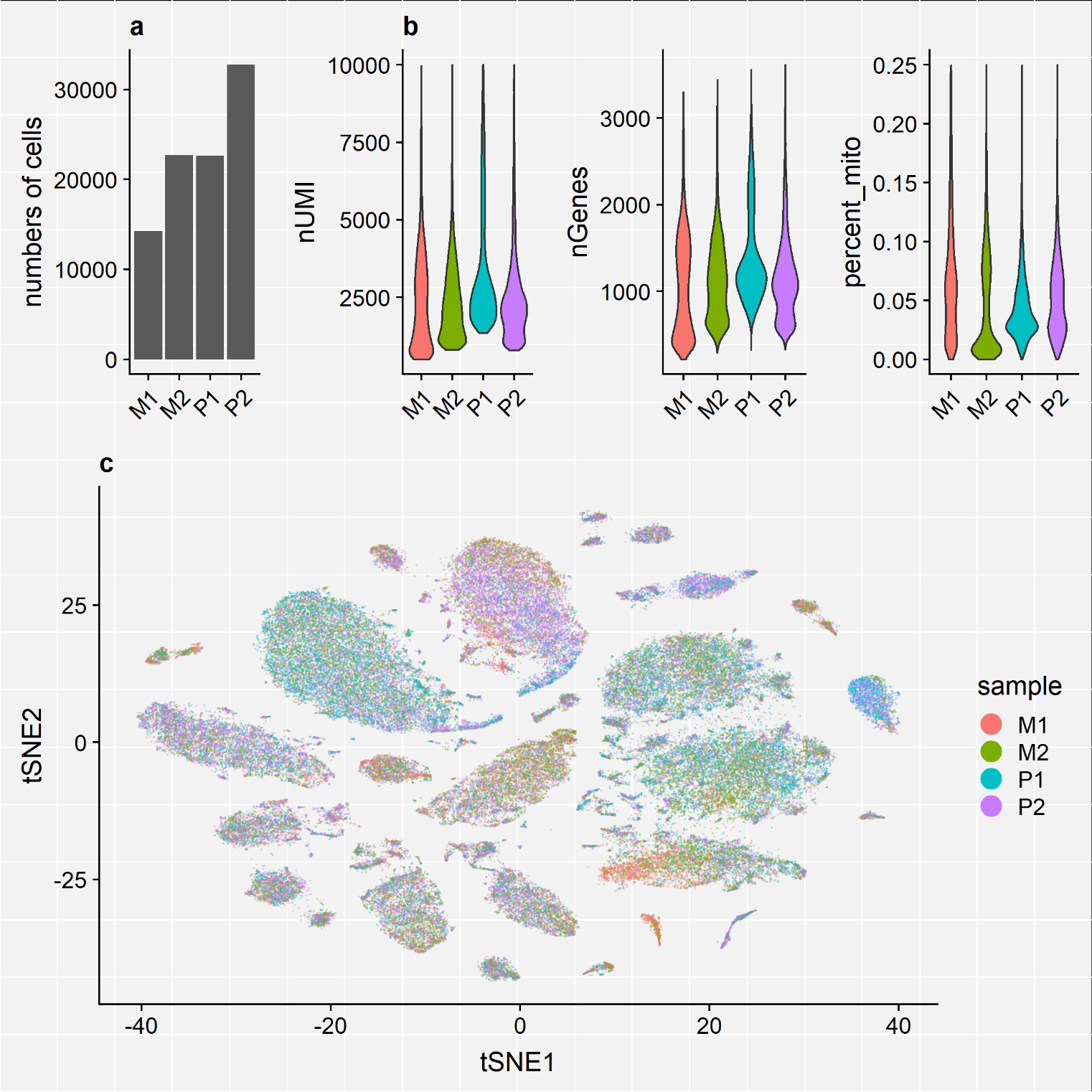
**

**Supplementary Fig. 1. Overview and quality control of scRNA-seq data**

(a) Bar graph showing the number of cells for the four filtered scRNA-seq samples (two samples for macular retina: M1 and M2, and two samples for peripheral retina: P1 and P2). (b) Violin plots showing the distribution of the number of UMIs per cell (left), number of genes per cell (middle) and percentage of mitochondrial genes per cell for the four scRNA-seq samples. (c) t-SNE projections of the scRNA-seq data. Color labels cells from different samples. The cells are randomly mixed, indicating batch effect was removed in clustering.


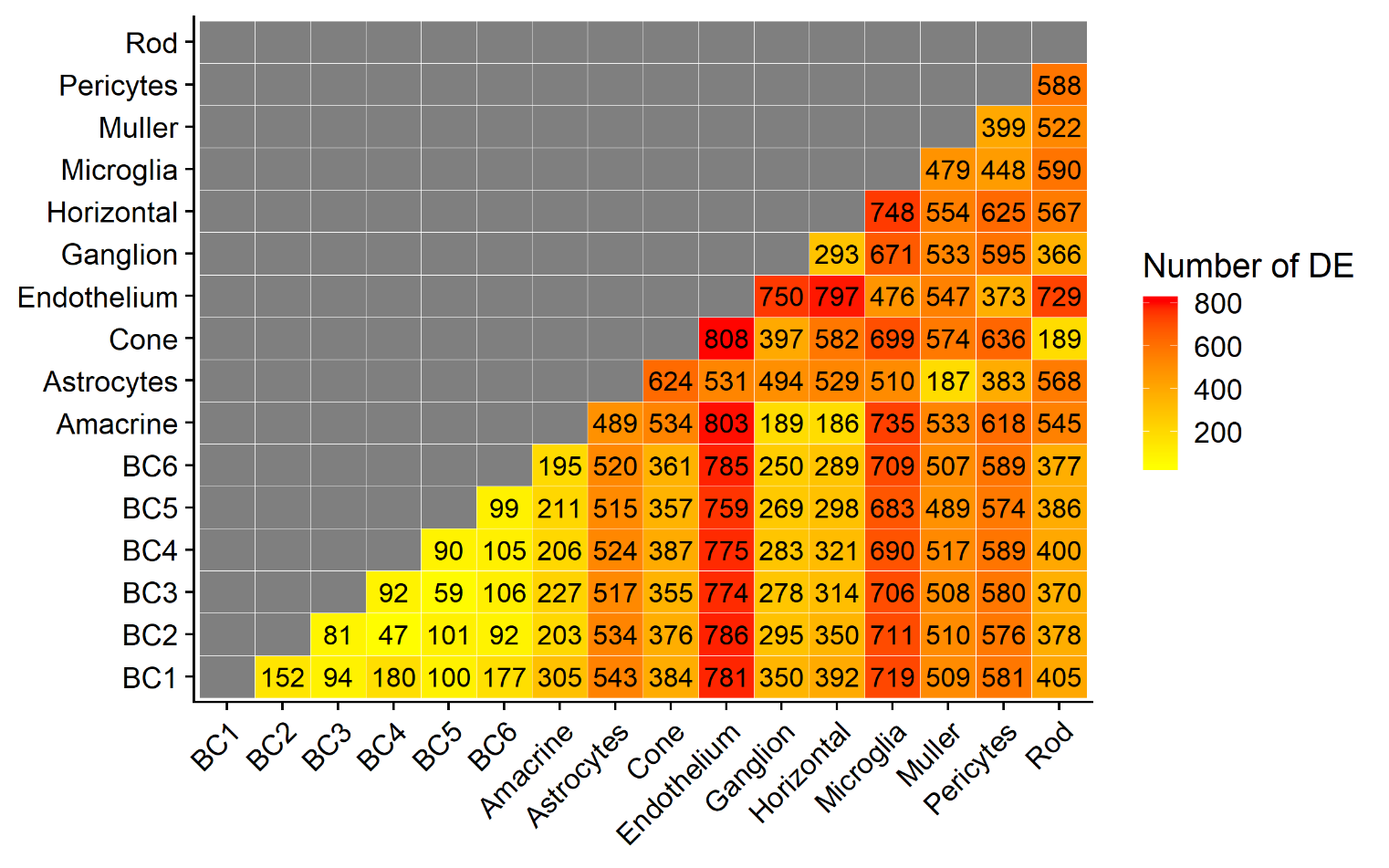


**Supplementary Fig. 2. Similarity in expression pattern across cell clusters**

The heatmap shows the number of significant DEGs detected between each pair of cell clusters. Sixteen cell clusters were identified using DESC and six of them were labeled as bipolar subtypes (BC1-BC6). The annotation in tiles show the exact number of significant DEGs for each pairwise differential expression analysis.

**
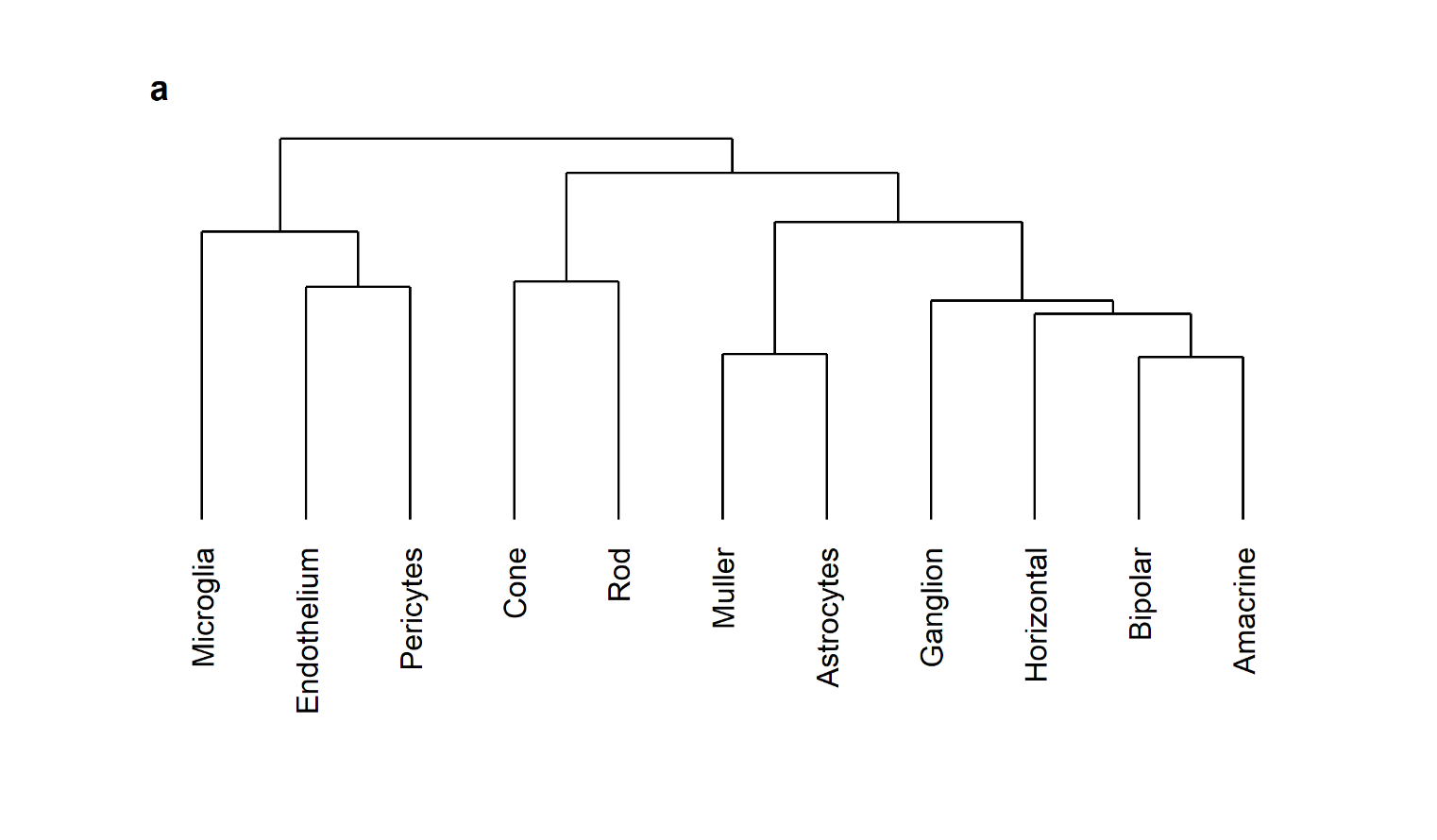
Supplementary Fig. 3. Dendrogram of identified cell types from scRNA-seq data**

The dendrogram shows the hierarchy of identified cell types. Hierarchical clustering analysis was performed on the mean expression for all genes across cells within each of the cell types.


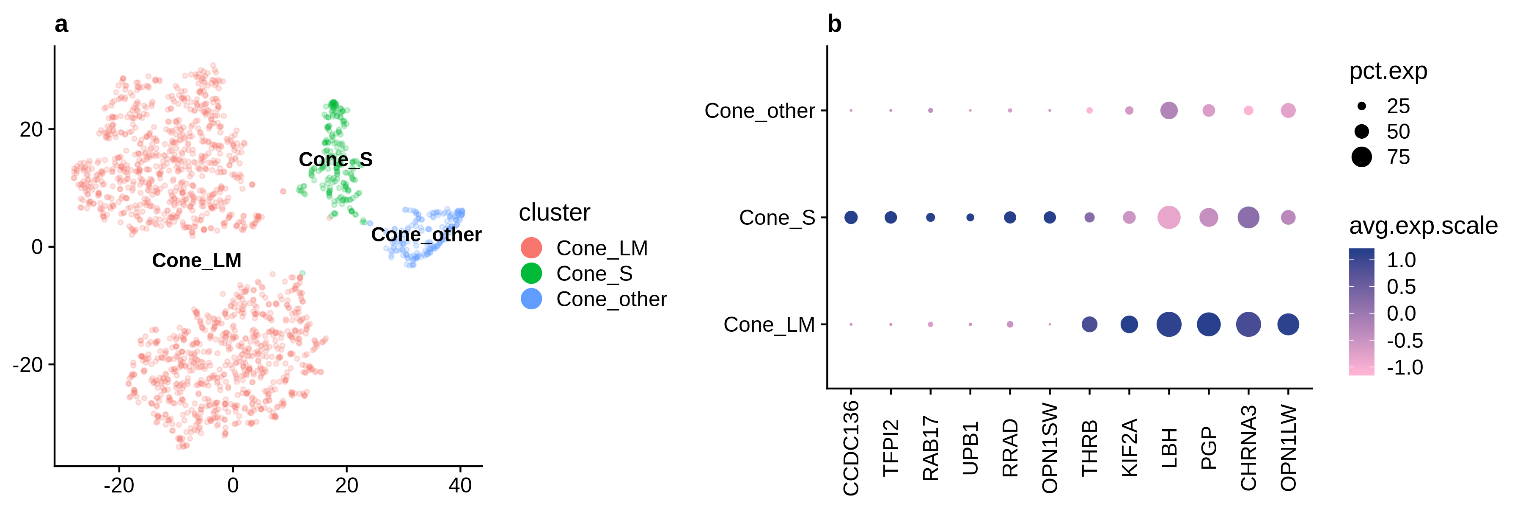


**Supplementary Fig. 4. Exploring cone subtypes.**

(a) t-SNE projections of all cone cells. Color labels cells from different cone subtypes. The Cone_other cluster was removed from the downstream analysis as we were unable to assign it to any known cone subtype. (b) Dot plots showing expression pattern of known cone subtype markers. The dot plot was generated using *Dotplot* in R Seurat package. The size of the dots represents percentage of cells that expressed gene markers while color shows average expression levels of gene markers.


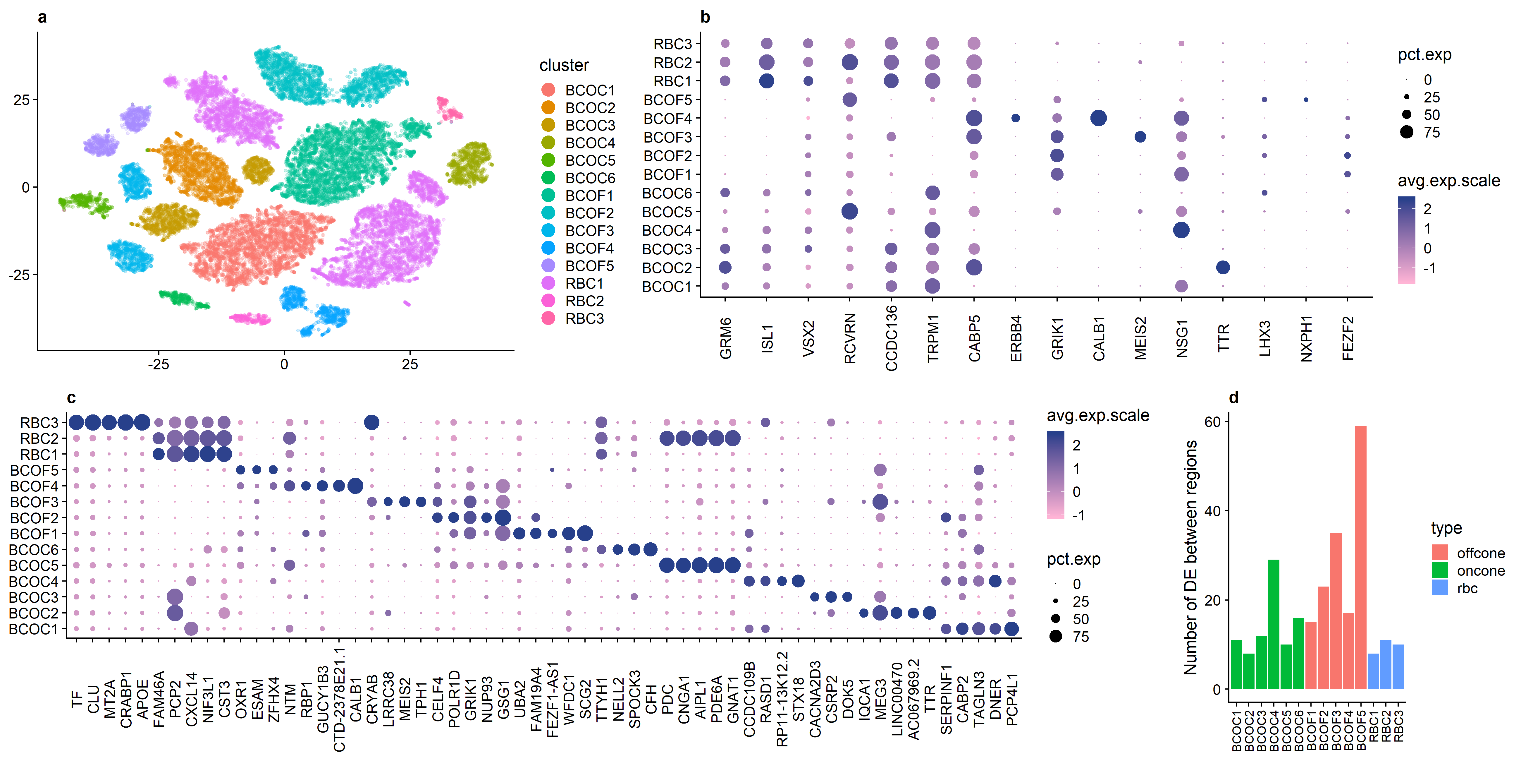


**Supplementary Fig. 5. Exploring bipolar subtypes.**

(a) t-SNE projections of all bipolar cells. Color labels cells from different bipolar subtypes. (b) Dot plots showing expression pattern of known cone subtype markers. (c) Dot plot shows the expression pattern of markers identified from different bipolar subtypes. The dot plot was generated using *Dotplot* in R Seurat package. The size of the dots represents percentage of cells that expressed gene markers while color shows average expression levels of gene markers. (d) Bar plot shows number of DEGs between two retina regions for each of the bipolar subtypes. Colors of the bars show 3 categories of biolar subtypes: offcone, oncone and RBC.


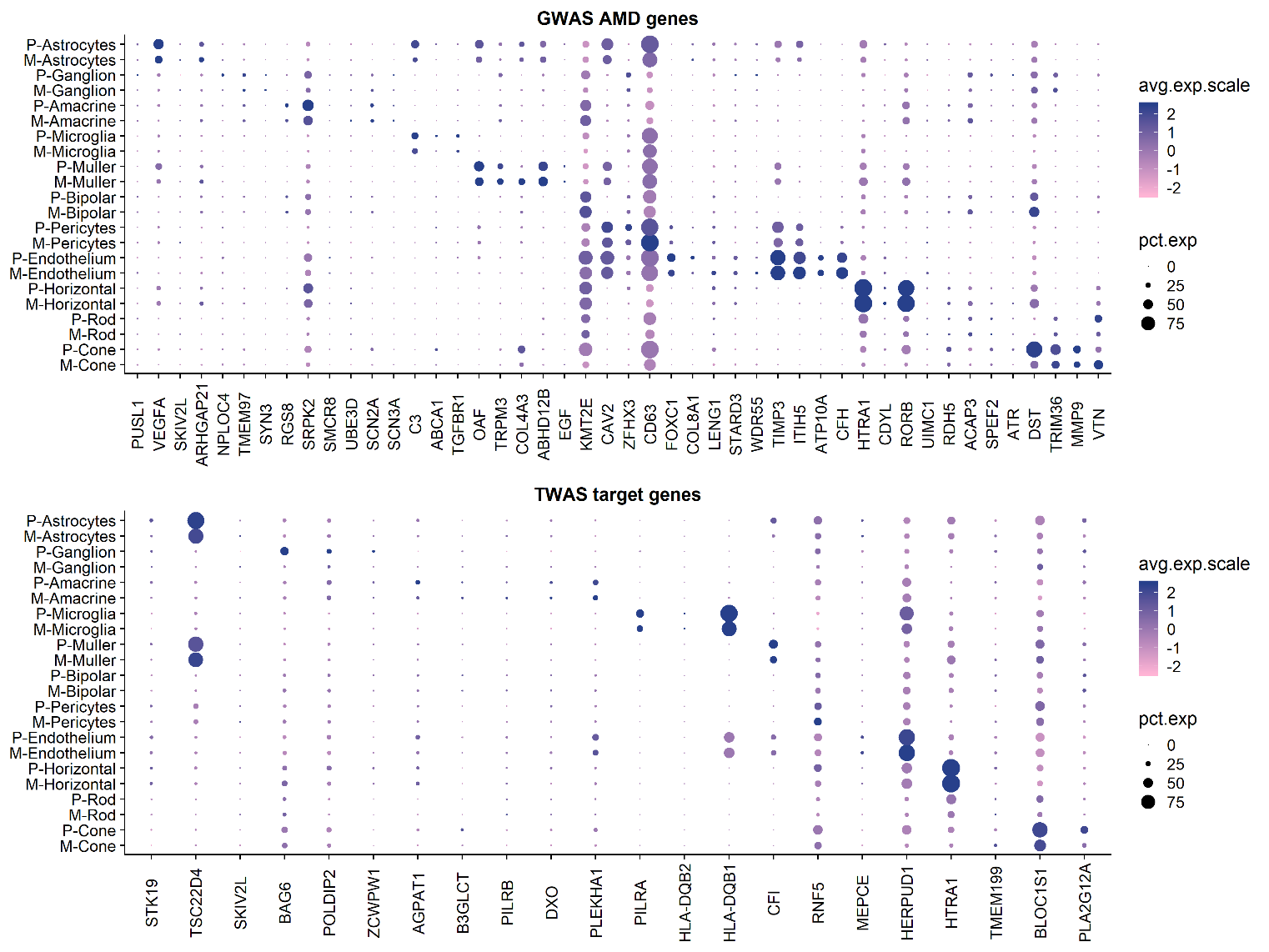


**Supplementary Fig. 6. Expression of AMD risk genes across cell types and retina regions.**

Dot plots showing expression patterns of AMD risk genes (top: 46 GWAS AMD genes, bottom: 22 TWAS target genes) across cell types and retina regions. The dot plot was generated using *Dotplot* in R Seurat package. The size of the dots represents percentage of cells that expressed gene markers while color shows average expression levels of gene markers.


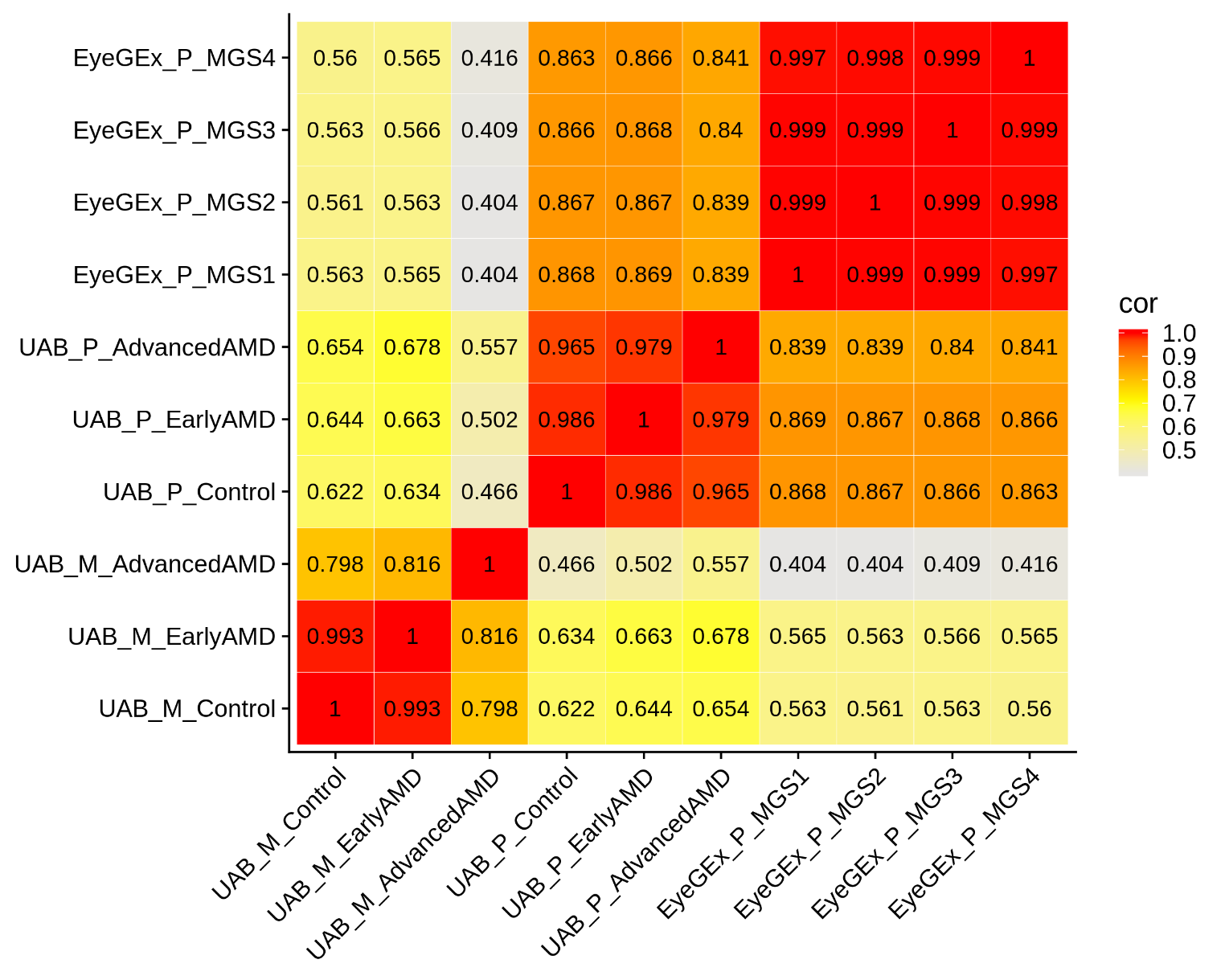


**Supplementary Fig. 7. Similarity of bulk RNA-seq data across datasets and conditions.**

The heatmap shows the similarity (Pearson correlation) in overall expression pattern across UAB and EyeGEx datasets and conditions. Pearson correlation was calculated using log scales read counts between each pair of samples. Only genes that existed in both datasets were considered in the analysis.


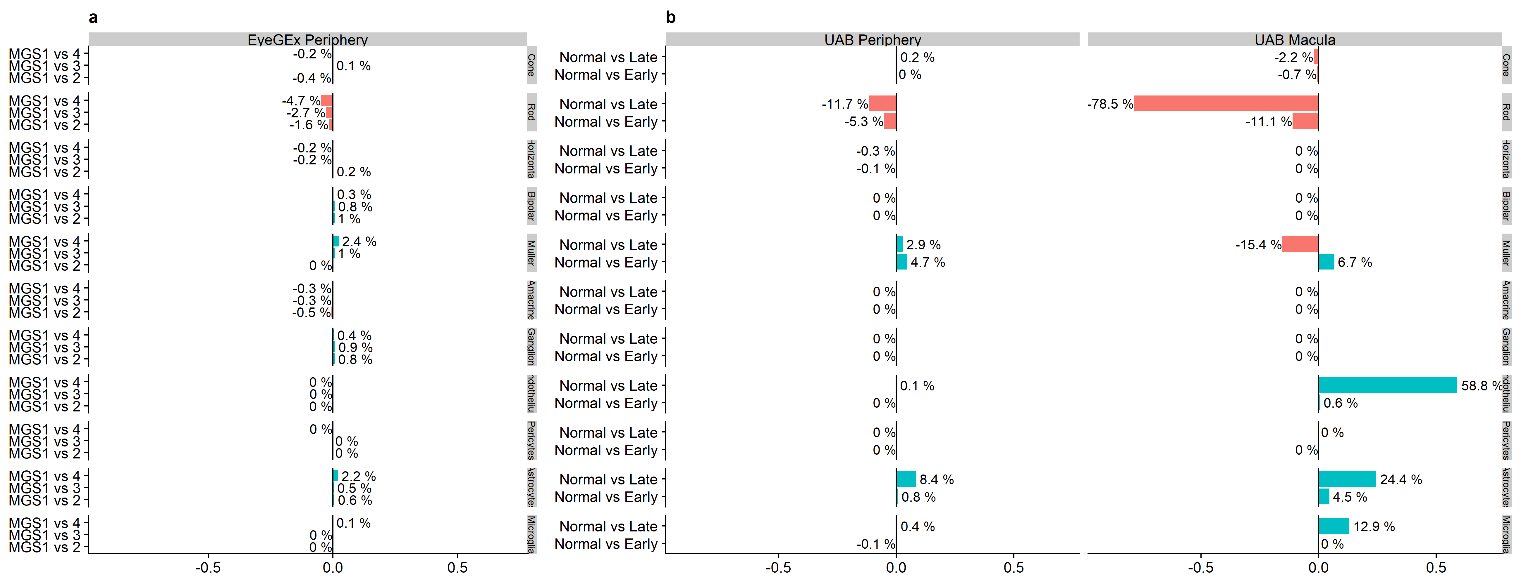


**Supplementary Fig. 8. Cell type proportion changes across AMD stages in EyeGEx dataset.**

The bar graphs show proportion changes of cell type across different AMD stages. Color shows the direction of the changes (red: decreasing, green: increasing). (a) Changes in cell type proportions in EyeGEx data when comparing MGS 2, 3, and 4 to MGS 1. (b) Changes in cell type proportions in UAB data when comparing early and late AMD to normal.

**Supplementary Data**

**Supplementary Data 1. List of known retina cell type marker genes.**

**Supplementary Data 2. Cell type gene markers detected from the scRNA-seq data.**

a. Cell type-specific genes

b. Cell type-specific genes - macula

c. Cell type-specific genes - periphery

**Supplementary Data 3. Cell type level differential expression between macula and periphery.**

**Supplementary Data 4. Cell subtypes exploration.**

a. Cone subtype differential expression between retina regions

b. Identified bipolar subtype gene markers

c. Bipolar subtype differential expression between retina regions

**Supplementary Data 5. Differential expression results for the UAB bulk RNA-seq data.**

a. Complete differential expression results

b. Selected genes for the enrichment analysis

**Supplementary Data 6. Kegg pathways for selected gene list**

**Supplementary Data 7. T-test result for cell type proportion changes**

**Supplementary Data 8. ctDEGs identified in the EyeGEx bulk RNA-seq data.**

a. ctDEGs identified between MGS2 vs. MGS1

b. ctDEGs identified between MGS3 vs. MGS1

c. ctDEGs identified between MGS4 vs. MGS1

**Supplementary Data 9. ctDEGs identified in the UAB bulk RNA-seq data.**

a. ctDEGs identified between Early AMD vs. Normal in macula

b. ctDEGs identified between Late AMD vs. Normal in macula

c. ctDEGs identified between Early and Late AMD vs. Normal in periphery
