# Supplementary material for: Implication of specific retinal cell-type involvement and gene expression changes in AMD progression using integrative analysis of single-cell and bulk RNA-seq profiling
Source: Sci Rep. 2021 Aug 2;11:15612. doi: 10.1038/s41598-021-95122-3 (PMC8329233; doi:10.1038/s41598-021-95122-3)
Supplement: Supplementary file 20 — Supplementary Information 20. [file 41598_2021_95122_MOESM20_ESM.docx]

**Supplementary Data Legned**

**Supplementary Data 1. List of known retina cell type marker genes.**

**Supplementary Data 2. Cell type gene markers detected from the scRNA-seq data.**

a. Cell type-specific genes

b. Cell type-specific genes - macula

c. Cell type-specific genes - periphery

**Supplementary Data 3. Cell type level differential expression between macula and periphery.**

**Supplementary Data 4. Cell subtypes exploration.**

a. Cone subtype differential expression between retina regions

b. Identified bipolar subtype gene markers

c. Bipolar subtype differential expression between retina regions

**Supplementary Data 5. Differential expression results for the UAB bulk RNA-seq data.**

a. Complete differential expression results

b. Selected genes for the enrichment analysis

**Supplementary Data 6. Kegg pathways for selected gene list**

**Supplementary Data 7. T-test result for cell type proportion changes**

**Supplementary Data 8. ctDEGs identified in the EyeGEx bulk RNA-seq data.**

a. ctDEGs identified between MGS2 vs. MGS1

b. ctDEGs identified between MGS3 vs. MGS1

c. ctDEGs identified between MGS4 vs. MGS1

**Supplementary Data 9. ctDEGs identified in the UAB bulk RNA-seq data.**

a. ctDEGs identified between Early AMD vs. Normal in macula

b. ctDEGs identified between Late AMD vs. Normal in macula

c. ctDEGs identified between Early and Late AMD vs. Normal in periphery
